# Biological and Biosimilar Medicines in Contemporary Pharmacotherapy for Metabolic Syndrome

**DOI:** 10.3390/pharmaceutics17060768

**Published:** 2025-06-11

**Authors:** Wiktoria Górecka, Daria Berezovska, Monika Mrozińska, Grażyna Nowicka, Monika E. Czerwińska

**Affiliations:** 1 Student Scientific Association, Department of Biochemistry and Pharmacogenomics, Medical University of Warsaw, 1 Banacha Str., 02-097 Warsaw, Poland; s083172@student.wum.edu.pl; 2 Department of Biochemistry and Pharmacogenomics, Medical University of Warsaw, 1 Banacha Str., 02-097 Warsaw, Poland; daria.berezovska@wum.edu.pl (D.B.); grazyna.nowicka@wum.edu.pl (G.N.); 3 Centre for Preclinical Research, Medical University of Warsaw, 1B Banacha Str., 02-097 Warsaw, Poland; 4 Provincial Infectious Diseases Hospital in Warsaw, 37 Wolska Str., 01-201 Warsaw, Poland

**Keywords:** biopharmaceutics, GLP-1 analogues, discovery, synthesis, pharmacokinetics, pharmacodynamics

## Abstract

The discovery of new drugs offers valuable alternatives, particularly for treating diseases that are resistant to existing therapies or involving complex, multi-organ conditions such as metabolic syndrome. Although treatment algorithms are generally well established and primarily based on synthetic pharmaceuticals, they are increasingly being supplemented by biological and biosimilar agents. This trend is particularly evident in the development and advancement of anti-diabetic and hypolipemic therapies. This review explores advances in the treatment of hypercholesterolemia and hypertriglyceridemia, elevated lipoprotein(a) [Lp(a)], diabetes, and obesity associated with metabolic syndrome. It focuses mainly on biopharmaceuticals such as proteins and nucleotide-based drugs (antisense oligonucleotides, small interfering RNA), but also on dipeptidyl peptidase-4 (DPP-4) inhibitors classified as incretin drugs along with glucagon-like peptide-1 (GLP-1) analogues. Due to the substantial role of SGLT-2 (sodium/glucose cotransporter 2) inhibitors in novel diabetes therapies, especially for managing cardiovascular risk, this group of compounds was also included in this review. Many clinical data in the field of effectiveness of biopharmaceuticals in metabolic disorders are provided. Therefore, in this review, we mainly include a brief history of drug development and approval, first synthesis and structure modifications, which relevantly influence pharmacokinetics, and safety. We provide only brief comparison of biological drugs with metformin and sulphonylureas derivatives. Databases such as PubMed, Scopus, and Google Scholar are searched for the period between 2000 and 2024.

## 1. Introduction

Metabolic syndrome (MS) is considered one of the main health problems in the world according to the World Health Organization. The estimated percentage of its global prevalence is about 25%. Metabolic syndrome is defined as an asymptomatic pathophysiological condition characterized by central obesity, high blood pressure, insulin resistance, dyslipidemia, and hyperglycaemia [1]. The first goal of MS treatment is to reduce risk factors for diabetes, hypertension, organ dysfunction, and cardiovascular diseases. However, economic development, increased interest in the so-called Western lifestyle, and lack of physical activity make this a growing challenge. The American Diabetes Association (ADA), the American Obesity Association, the Obesity Society, the European Society of Hypertension, the American Heart Association, and the European Atherosclerosis Society provide numerous guidelines for treating and position statements each year [2,3,4]. Clinical treatment protocols are progressively incorporating biological and biosimilar agents to enhance therapeutic efficacy and broaden treatment options. Such evolution is particularly evident in the development of anti-diabetic drugs. A few decades ago, only metformin, biguanide, sulphonylureas derivatives, *α*-glucosidase inhibitors, and, since the last decade of the 20th century, thiazolidinediones (rosiglitazone and pioglitazone) were available for the treatment of type 2 diabetes (T2D) [5]. Diabetes treatment, particularly in the form of more comfortable drugs for patients, will likely differ significantly from current standards unless the transplantation of induced pluripotent stem-cell-derived islets becomes possible in a larger group of patients following the recent one-case success [6].

Apart from a wide range of synthetic drugs, the history of pharmacy is littered with examples of molecules discovered in nature, such as morphine-type alkaloids or the first statins (mevastatin, lovastatin, pravastatin), or isolated from fungi like *Penicillium*, *Aspergillus*, *Monascus*. Among antidiabetic drugs, SGLT-2 (sodium/glucose cotransporter 2) inhibitors are agents originating from plant compounds such as phlorizin. However, biological or biosimilar drugs deriving from living organisms through biotechnological procedures have recently attracted even greater interest. Biopharmaceuticals, also referred to as biological medicinal products, are therapeutic agents that are manufactured in, extracted from, or semi-synthesized in biological sources. They are used in the prevention, diagnosis, and treatment of cancer, autoimmune diseases, and various other diseases [7]. Biopharmaceuticals, comprising protein and nucleotide-based therapeutics, including antibodies, interleukins, and vaccines, represent a transformative approach in the management of chronic metabolic and cardiovascular diseases, including dyslipidemia, elevated lipoprotein(a) [Lp(a)], and diabetes mellitus. These conditions are interlinked by their contributions to the global burden of morbidity and mortality, primarily through cardiovascular complications. Traditional pharmacotherapies, such as statins and metformin, have laid the foundation for disease management but often fail to adequately address risk, particularly in patients with elevated Lp(a) or diabetes-related cardiovascular comorbidities [8,9].

This review explored the advancements in treating diabetes, dyslipidemia, elevated Lp(a), and obesity. We aimed to emphasize their historical development, first synthesis and structure modifications, pharmacokinetics, and safety. The molecular mechanisms of action, therapeutic efficacy, and clinical outcomes were just briefly described. We were particularly focused on biopharmaceuticals such as protein or peptide-based drugs, including insulin, glucagon-like peptide-1 (GLP-1) receptor analogues, proprotein convertase subtilisin/kexin type 9 (PCSK9) inhibitors, and monoclonal antibodies (e.g., alirocumab and evolocumab). As far as nucleotide-based drugs are concerned, antisense oligonucleotides (ASOs) and small interfering RNA (siRNA), represented by pelacarsen and inclisiran, respectively, were included. However, due to the substantial role of SGLT-2 inhibitors in the novel diabetes therapy in cardiovascular burden, this group of compounds was also included in this review. A comprehensive literature search was conducted using databases such as PubMed, Scopus, and Google Scholar, encompassing publications from 2000 to 2024. Certain individual publications predate the specified timeframe due to the inclusion of sources referenced for specific procedures and information.

## 2. Antidiabetic Drugs

### 2.1. Insulin

In 1890, Mering and Minkowski discovered the link between the pancreas and diabetes. In one of their experiments on a dog whose pancreas was completely removed, they observed the occurrence of polyuria due to glucosuria resulting from hyperglycaemia [10,11]. In 1921, Banting and Best, using crude canine pancreas extract for the first time, successfully reduced hyperglycaemia in a dog after the pancreas was removed [10]. In 1922, a purified insulin extract was administered to a 14-year-old boy with type 1 diabetes (T1D) with spectacular success [10,11]. For many years, insulin for diabetes treatment was derived from animal pancreas because of its structural and functional similarity to human insulin. However, its long-term use led to immune intolerance, contributing to the development of numerous complications in patients with diabetes [12].

In 1978, researchers at the City of Hope and Genentech successfully established a methodology for producing biosynthetic human insulin utilizing recombinant DNA technology. After manipulating the genes encoding the A and B chains of insulin, they programmed an *Escherichia coli* bacterium to mass produce the hormone [13,14]. In 1982, the U.S. Food and Drug Administration (FDA) approved Humulin, the first biosynthetic insulin. The introduction of this technology revolutionized the treatment of diabetes and sparked the development of modern insulin analogues, such as the fast-acting Humalog (1996) and the long-acting Lantus (2000) [13].

Pluripotent stem cells have recently emerged as a promising source of cells for cellular replacement therapies, such as islet transplantation. The first case of autologous transplantation of chemically induced pluripotent stem cell-derived islets into a patient with T1D under immunosuppressive therapy has been reported. The successful experiment led to sustained insulin independence and restoration of glycemic control [6], opening up new possibilities for treating diabetes.

#### 2.1.1. Insulin Structure and Its Modifications

**Figure 1:**
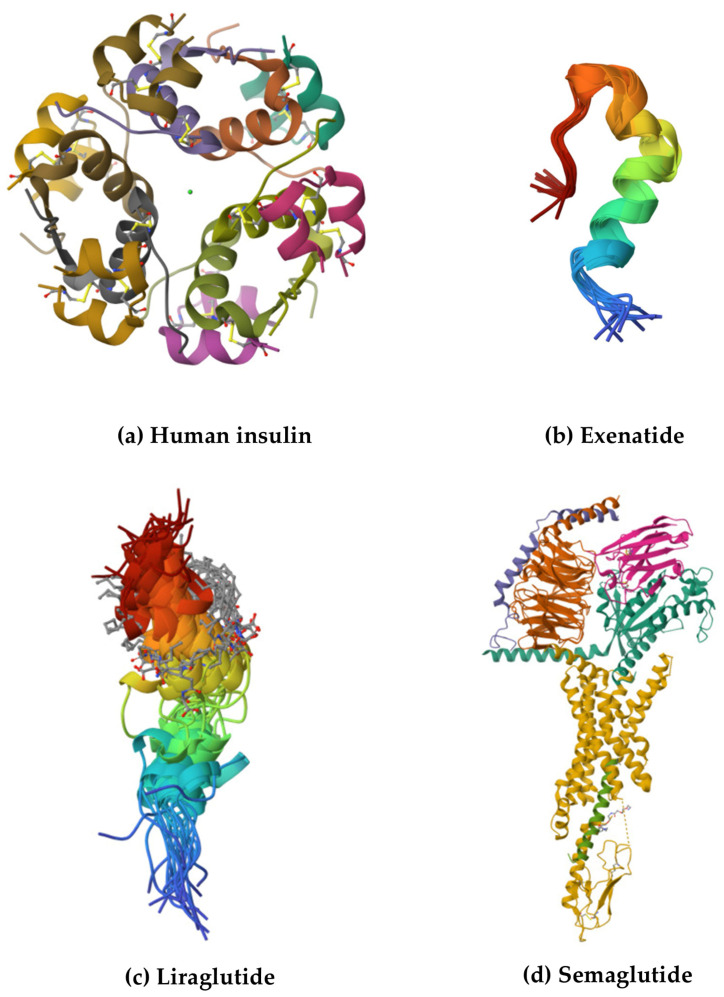
The structures of selected biological drugs used in the treatment of T2D: (**a**) crystal structure of human zinc insulin at pH 5.5, (**b**) Trp-cage fortified Tc5b-exenatide chimera (Ex-4-Tc5bQR) at 277K, (**c**) liraglutide, and (**d**) semaglutide-bound glucagon-like peptide-1 receptor in complex with Gs protein. The structures and their descriptions are available in the Protein Data Bank [17].

Insulin is a peptide hormone composed of two polypeptide chains, designated as the A and B chains, linked by two interchain disulfide bonds. The A chain consists of 21 amino acid residues, while the B chain comprises 30 amino acid residues [12]. Endogenous insulin is formed by the enzymatic breakdown of proinsulin, which is split into two molecules: insulin and C-peptide [15]. Native insulin forms dimers, which in the presence of zinc ions combine to form hexamers (Figure 1), facilitating the storage in β-cell vesicles. After exocytosis and dilution, the hexamers rapidly dissociate into active monomers [16].

The structure of animal insulin has slight but potentially significant differences compared to human insulin. Porcine insulin differs from human insulin by a single amino acid substitution, in which alanine replaces threonine at the carboxy-terminus of the B chain (position B30). In contrast, beef insulin has two additional modifications in the A chain sequence, where threonine and isoleucine at positions A8 and A10 are substituted with alanine and valine, respectively. Despite these variations, porcine insulin shares an almost identical amino acid sequence with human insulin [18]. It is worth noting that biological drugs produced by living organisms are changeable. This characteristic, known as microheterogeneity, can be detected in biopharmaceuticals without compromising their established safety and efficacy profiles [19,20].

Currently, several types of insulins are available, classified based on their purpose and duration of action. Basal insulins include intermediate-acting human insulin and long-acting insulin analogues [21]. Mealtime insulins consist of short-acting regular human insulin, rapid-acting insulin analogues, and ultra-rapid-acting insulin analogues [14,21,22], as well as pre-mixed insulins [21]. The summary of insulin types and formulations is shown in Table 1 and Table 2.

In insulin glargine, a long-acting type of insulin, two arginine residues are added to the C-terminus of the B chain, and asparagine at position 21 of the A chain is replaced with glycine. This structural modification enhances the chemical stability of the insulin by preventing the deamidation of asparagine, which could otherwise affect its properties [21].

Unlike human insulin, detemir does not have a threonine at position B30 in its structure, and the lysine at position B29 has been modified by the attachment of myristic acid, a 14-carbon fatty acid. This structural modification enhances the self-association of insulin detemir and facilitates its reversible binding to albumin, thereby conferring prolonged pharmacokinetic properties [22].

The long-acting insulin analogue, degludec, retains the same amino acid sequence as human insulin, except for the absence of threonine at position B30 and the attachment of a 16-carbon fatty acid to lysine at position B29 via a linker from glutamic acid. These modifications cause degludec to precipitate in the subcutaneous tissue after injection, forming a deposit that breaks down gradually at a predictable rate [28]. According to data provided by the ADA, ultra-long-acting insulin such as glargine U-300 (Toujeo) can also be distinguished. It reaches the bloodstream in 6 h, does not peak, and the action lasts approximately 36 h or longer [29].

Insulin lispro is an analogue of insulin in which the natural sequence of amino acids in the B-chain at positions 28 and 29 is reversed [30]. In the case of insulin aspart, the amino acid proline located in the B chain is substituted with aspartic acid [31]. This modification produces an insulin molecule with a diminished ability to self-associate into hexamers [30]. As a result, insulins lispro and aspart are absorbed more rapidly after subcutaneous injection, making them particularly effective for managing postprandial blood glucose levels.

Insulin glulisine is a rapid-acting insulin analogue that differs structurally from human insulin through two key amino acid substitutions: lysine replaces asparagine at position B3 and glutamic acid takes the place of lysine at position B29. Chemically, it is known as 3B-lysine-29B-glutamic acid human insulin [32]. Glulisine is a zinc-free insulin analogue designed for rapid absorption. Crystallographic studies reveal that zinc is essential for hexamer formation. However, modifications at B3 and B29 in glulisine prevent the formation of both dimers and hexamers, making it one of the most effective rapid-acting insulin analogues [32].

Faster-acting insulin aspart is a modified formulation of insulin aspart with nicotinamide and arginine, enhancing stability and accelerating absorption after subcutaneous injection. This adjustment aims to improve postprandial glycemic control by imitating a more natural insulin response [23].

Ultra-rapid insulin lispro is a modified formulation of insulin lispro that contains two additional excipients. Treprostinil acts topically to cause vasodilation, while citrate increases vascular permeability. These changes contribute to faster insulin absorption [33].

In summary, structural modifications of insulin in analogues, such as changes in the amino acid sequence or the attachment of fatty acids, allow its action to be tailored to therapeutic needs. With these modifications, insulins can act faster, longer or more predictably, improving the effectiveness of diabetes treatment and the quality of patient life.

#### 2.1.2. Mechanism of Action

In healthy people, plasma glucose levels remain within a narrow range (3.5–7.0 mmol/L; 70–99 mg/dL) throughout the day. Following a meal, blood glucose levels typically increase within 30 to 60 min, reach a peak, and return to baseline values within 2 to 3 h. In fasting, the rates of glucose production and utilization are balanced. Postprandial glucose homeostasis is primarily maintained through insulin secretion and the concomitant suppression of glucagon release [34]. 

**Figure 2:**
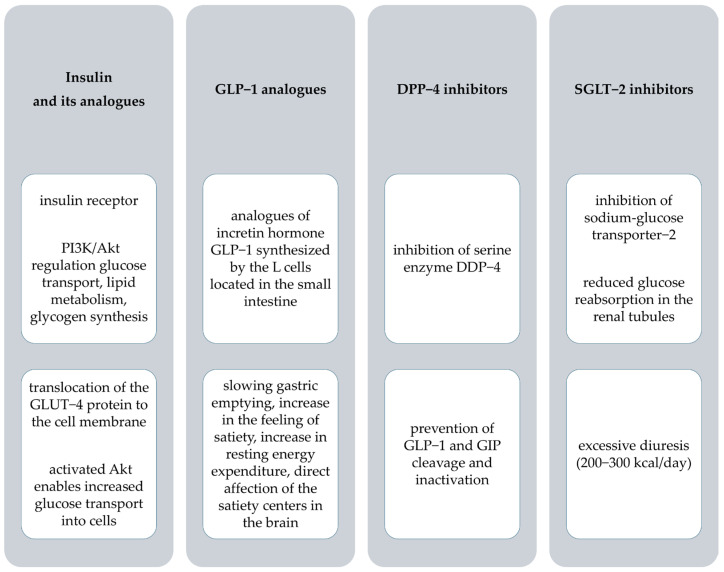
The mechanism of action and pharmacological effects of anti−diabetic drugs. PI3K, phosphatidylinositol 3 kinase; Akt, protein kinase B; GLUT−4, glucose transporter−4; GLP−1, glucagon-like peptide−1; DPP−4, dipeptidyl peptidase−4; GIP, glucose−dependent insulinotropic polypeptide; SGLT−2, sodium−glucose transporter−2.

Insulin reduces blood glucose concentrations (Figure 2), stimulating its uptake by skeletal muscle and adipose tissue while increasing lipogenesis and promoting protein synthesis [35].

Insulin analogues, like human insulin, bind to insulin receptors on the surface of cells, allowing glucose to be transported into the cells and lowering blood glucose levels. Due to structural modifications, analogues differ from human insulin, allowing them to act faster or longer, enabling better-tailored therapy and improved glycemic control [36].

#### 2.1.3. Pharmacokinetics of Insulins

##### Basal Insulins

NPH (Neutral Protamine Hagedorn) insulin is a suspension preparation composed of insulin, protamine, and zinc. The prolonged effect is due to its crystalline structure. After subcutaneous administration, insulin is released gradually from the precipitate formed. The rate of insulin release depends on several factors, including the presence of zinc ions, which stabilize the precipitate structure, and additives such as protamine and phenolic derivatives, which modify the properties of the precipitate and affect the duration of insulin action [37]. The onset of NPH insulin typically occurs within 2 to 4 h after subcutaneous injection. The peak effect is usually observed between 4 and 10 h post injection (Table 1). The action can last from 8 to 16 h, depending on individual factors such as physical activity, diet, and the patient’s overall health [38]. Due to its high variability of action, NPH insulin is associated with a higher risk of both hypoglycemia and hyperglycemia compared to modern insulin analogues [24,39].

The long-acting soluble insulin analogues demonstrate pharmacokinetic and pharmacodynamic properties that are more physiologically consistent than those of NPH insulin. These analogues provide a more stable action profile with a prolonged duration of effect, especially after several days of continuous use. Moreover, reduced within-subject variability and minimized fluctuations were observed. These advantages contribute to a lower risk of nocturnal hypoglycemia in individuals with T1D when compared to NPH insulin [40].

The pharmacokinetic characteristics of insulin glargine indicate that it begins to take effect approximately 2 h following subcutaneous administration. In contrast to NPH insulin, it does not show a peak of action. This results in a consistent, stable release of insulin throughout the day. The duration of its action extends for around 24 h, providing long-lasting blood glucose control with a single daily injection [41]. Rosenstock et al. demonstrated that treatment with glargine is associated with a lower risk of hypoglycemia compared to the use of NPH insulin. Although the total daily insulin doses were similar in both cases, patients using glargine experienced approximately 29% fewer episodes of hypoglycemia compared to those treated with NPH insulin [42].

Glargine U-300 (Gla-300) is designed to improve the stability of action and prolong the duration of basal insulin activity. Due to its higher concentration (300 units/mL), this insulin formulation forms a more compact subcutaneous depot upon administration, contributing to a slower rate of absorption and a more stable pharmacodynamic profile. This unique pharmacological approach minimizes the risk of fluctuations in insulin concentration, which is important for reducing the risk of hypoglycemia [43,44]. The onset of action of Gla-300 is approximately 6 h, resulting from the gradual release of insulin from the depot. The pharmacokinetic profile also demonstrates a longer duration of action of approximately 36 h, indicating that the insulin remains active significantly longer than Gla-100 [44]. The time to reach 50% of exposure (T_50%_) of Gla-300 was significantly longer than Gla-100, highlighting the more stable release of insulin from subcutaneous deposition. In addition, a swing index of less than 1 for Gla-300 indicates less variation in serum insulin concentration, resulting in a more even profile of action [43].

Insulin detemir has a relatively rapid onset of action, which depends on the dose administered. The average onset of action ranges from 1 to 2 h. Time to peak concentration (T_max_) is reached between 6 and 12 h, irrespective of the dose administered. These findings indicate uniform absorption of the formulation, which contributes to the stability of its therapeutic effect over time [24,45]. The average duration of action of insulin detemir is approximately 17.5 h. However, this may vary depending on the administered dose, potentially extending its effect to nearly 24 h [40]. The prolonged action profile, combined with lower inter-patient variability compared to traditional insulins such as NPH, promotes a reduced risk of hypoglycemia and improved glycemic control [45]. Porcellati et al., presented pharmacokinetic and pharmacodynamic results suggesting that in T1D patients, detemir insulin is most effective as basal insulin twice daily [40].

Insulin degludec is an ultra-long-acting insulin with a stable pharmacokinetic profile (peak-free) and a duration of action of more than 42 h. Studies in patients with T1D have shown that insulin degludec reduces the risk of nocturnal hypoglycemia by 25% compared to insulin glargine, whereas the incidence of daytime hypoglycemia was comparable between the two groups [46]. In contrast, Heise et al., demonstrated that insulin degludec, at steady state, exhibited a more predictable glucose-lowering profile and facilitated more precise dose adjustment, which may contribute to a reduced risk of hypoglycemia [47].

##### Mealtime Insulins

Insulin therapy aims to mimic physiological insulin secretion. Short-acting insulins reduce postprandial glycemia, minimizing the risk of hypoglycemia. The dose of insulin used at meals is changeable depending on several factors, such as the amount of carbohydrates, the glycemic index of the meal, and individual insulin sensitivity. As the regular human insulin (RHI) dose increases, there is not only an increase in insulin exposure and metabolic activity but also an increase in the duration of insulin action, which increases the risk of late postprandial hypoglycemia [48]. Human neutral insulin exhibits a relatively rapid onset of action. It begins to act within 30 min following subcutaneous injection. The maximum plasma concentration is reached between 2 and 4 h after drug administration. The total duration of action of RHI is approximately 5–8 h [24].

Rapid-acting insulin analogues (RAIA) were developed to address the problem of delayed subcutaneous absorption of RHI. Rapid-acting insulin analogues are effective in limiting postprandial glucose spikes, reducing the 2-h postprandial plasma glucose concentration, and the late hypoglycemia compared to RHI. Rapid-acting insulin analogues are used before meals, often in combination with basal insulins or oral medications, to mimic the natural insulin response after eating. These medications are characterized by rapid absorption, shorter duration of action, and better glucose control after meals [49]. The data on the action onset, the time to achieve maximum concentration, and the duration of action are consistent across several sources [23,24,25,26,27]. They are displayed in Table 1.

##### Pre-Mixed Insulins

Pre-mixed insulins are a combination of short-acting insulin or rapid-acting analogue with intermediate-acting insulin in specific proportions (Table 2). Medications allow simultaneous control of fasting and postprandial glucose levels with a single injection. Examples include biphasic human insulins (e.g., 30/70 and 50/50), analogues such as biphasic insulin aspart (e.g., 30/70 and 50/50), as well as biphasic insulin lispro (25/75 and 50/50). Compared to self-mixed insulins, ready-to-use mixtures offer greater dosing accuracy, efficacy, and convenience, which promotes better adherence to therapy and more effective long-term diabetes control [50].

The effect of pre-mixed insulin starts within 15–60 min after injection and can last between 10 and 16 h. The peak of activity varies depending on the types of insulin used [51].

### 2.2. Glucagon-like Peptide-1 Analogues

Glucagon-like peptide-1 analogues are a group of drugs used to treat T2D and obesity, and their use should always be combined with diet and lifestyle changes. These drugs are synthetic compounds that mimic the functions of natural GLP-1: stimulate insulin secretion by β-pancreatic cells [52,53] and block glucagon secretion by α-pancreatic cells [54]. They work in a blood glucose-dependent manner, suggesting that these drugs remain inactive at low glucose levels, significantly reducing the risk of hypoglycemia [52,53]. Analogues of GLP-1 have multidirectional therapeutic effects. In addition to improving glycemic control, they contribute to weight reduction, have beneficial effects on lipid metabolism, and help to lower blood pressure. Furthermore, the results of numerous clinical trials indicate their ability to reduce the risk of cardiovascular complications, making them particularly valuable in treating patients with T2D [52]. In 2022, the ADA recommended GLP-1 agonists as a first-line therapy for T2D, particularly in patients with atherosclerotic cardiovascular disease or obesity [55].

The first FDA-approved GLP-1 analogue, exenatide (marketed as Byetta^®^), was introduced in 2005 [56], and the next GLP-1 receptor agonists (GLP-1RAs) included liraglutide (marketed as Victoza^®^) [57] and lixisenatide (marketed as Lyxumia^®^) [57,58]. In 2011, the European Medicines Agency (EMA) approved exenatide (marketed as Bydureon^®^), which became the first long-acting GLP-1 analogue [59]. Following this, albiglutide (marketed as Eperzan^®^) was launched in 2014, but now it is withdrawn [60], and the next drug introduced was dulaglutide (marketed as Trulicity^®^) [61]. In 2018, semaglutide (marketed as Ozempic^®^), another long-acting GLP-1RA, was introduced to the market [62]. The FDA approved the above drugs for use in T2D. Some of them, including semaglutide and liraglutide, are also used for treating obesity. However, GLP-1 analogues have not been approved for treating T1D [52]. The structures of selected GLP-1 analogues are shown in Figure 1. Only the structures available in the Protein Data Bank are displayed [17].

#### 2.2.1. Drug Manufacturing

Exenatide is a synthetic analogue of the GLP-1 peptide found in the saliva of the *Gila monster* lizard [63]. The synthesis of exenatide, based on the solid-phase peptide synthesis (SPPS) method, was the subject of patent no. US20130289241A1. It is based on the sequential attachment of amino acids to a polymer resin, according to a predetermined peptide sequence. The process begins with preparing the resin, to which the first protected amino acid residue, such as Fmoc-Ser(tBu)-OH, is attached. Then, the Fmoc-protective groups are removed step by step, and more amino acids are condensed, building the polypeptide chain from the C-terminus to the N-terminus. Once the synthesis is complete, the peptide is detached from the resin in a controlled manner, resulting in a high-purity product [64].

Similarly, the synthesis of lixisenatide using the aforementioned SPPS method was patented (patent no. CN103709243A). The first step is to prepare a suitable peptide resin. For this purpose, Fmoc-Lys(Boc)-OH is coupled to the resin, leading to Fmoc-Lys(Boc)-NH2-resin, which is the starting point for further synthesis. This is followed by a step-by-step elongation of the peptide chain, in which successive amino acids are attached according to the lixisenatide sequence. Once the peptide fragments are synthesized, they are joined together to form a complete sequence. The peptide is then cleaved using appropriate reagents, and the crude product is purified chromatographically. This synthesis method guarantees high purity and quality of lixisenatide [65].

Dulaglutide, known as LY2189265, is a fusion protein that links a GLP-1 analogue to the Fc domain of IgG4 immunoglobulin. Its synthesis involves several steps. First, a mutant GLP-1, resistant to degradation by the DPP-IV enzyme, was engineered and fused to the Fc domain to increase the duration of action. The genetic construct was introduced into HEK 293-EBNA cells, where the protein was produced under controlled conditions. The fusion protein was then purified using a HiTrap Protein A column (affinity chromatography) and Superdex 200 (gel permeation chromatography), yielding a high-purity product. The purified GLP-1-Fc fractions were analyzed by SDS-PAGE (SDS-polyacrylamide gel electrophoresis) and mass spectrometry. A suitable linker between GLP-1 and Fc was also introduced to improve the biological activity of the molecule, and immunogenic epitopes were removed to reduce the risk of immune reactions [66].

Semaglutide was designed based on human GLP-1, introducing two key changes in the amino acid sequence: substitution of α-aminoisobutyric acid (Aib) at position 8 and arginine at position 34. Semaglutide was synthesized using a standard SPPS method with the Fmoc strategy. The lysine at position 26 was modified by coupling to a C18 fatty diacid using a γ-glutamic acid-2xOEG [oligo(ethylene glycol)] linker. After synthesis, the Mtt (4-methyltrityl) protecting group was removed, and the peptide was detached from the resin using a mixture of trifluoroacetic acid (TFA), water and triisopropylsilane, followed by purification by RP-HPLC. The purity and identity of the semaglutide were confirmed by UPLC and LC-MS techniques, achieving a purity of at least 95%. This modification provided semaglutide with adequate stability and extended duration of action [63].

Liraglutide is an analogue of human GLP-1 designed to bind to human albumin via a fatty acid and a linker covalently attached to the peptide backbone [63]. Liraglutide was synthesized using Fmoc/tBu solid-phase peptide synthesis method (patent no. US11066439B2). The process involved the stepwise attachment of successive amino acid residues to the Fmoc-basedresin. After the sequence was completed, the peptide was detached from the product using a reagent mixture containing TFA [67]. 

Tirzepatide, also known as LY3298176, is a 39-amino acid peptide designed as a dual agonist for GIP (glucose insulinotropic polypeptide) and GLP-1 receptors. Its chemical structure includes unique elements, such as non-coding amino acids at positions 2 and 13 (α-aminoisobutyric acid) and an amidated C-terminus. A key feature of tirzepatide’s design is the application of acylation technology, which facilitates reversible binding to albumin. This results in a long-lasting pharmacokinetic profile. The process of synthesising tirzepatide was based on traditional peptide chemistry methods. After synthesis, the peptide was dissolved in phosphate buffer (PBS) to prepare it for further studies. The tirzepatide shows high affinity for both receptors (Ki for GIP: 0.135 nM, GLP-1: 4.23 nM) [68].

#### 2.2.2. Mechanism of Action and Pharmacological Effects

Glucagon-like peptide-1 is a type of incretin hormone [54]. The receptors of GLP-1 are found in many organs, namely the heart, pancreas, lungs, brain, stomach, and gastrointestinal tract [69]. Diverse receptor localization explains the multidirectional effects of GLP-1 protein (Figure 2).

Analogues of GLP-1 beneficially affect the heart and cardiovascular system. They have been shown to help reduce cardiometabolic risk factors. All GLP-1 analogues lower systolic blood pressure, while the effect on diastolic pressure depends on the specific drug. However, most of them tend to increase diastolic blood pressure.

In addition, GLP-1 analogues help improve lipid profile by lowering total plasma cholesterol, low-density lipoprotein (LDL) and very low-density lipoprotein (VLDL), and plasma triglycerides while increasing HDL-cholesterol levels [70,71,72,73].

The use of GLP-1 analogues was found to be associated with an increase in heart rate. In studies on the effects of individual drugs, an increase in heart rate was observed in the study group compared to placebo: for exenatide by 2.51 bpm (beats per minute), for lixisenatide by 0.4 bpm, for liraglutide by 3 bpm, for semaglutide at 0.5 mg by 2 bpm, at 1 mg by 2.5 bpm, and for dulaglutide by 1.87 bpm [70,73,74,75,76].

Some GLP-1 analogues, such as dulaglutide, semaglutide, and liraglutide, have been proven to reduce cardiovascular risk [70,74,75]. A study involving patients with T2D and elevated cardiovascular risk demonstrated that treatment with dulaglutide, liraglutide, or semaglutide was associated with a significant reduction in the composite risk of cardiovascular death, non-fatal myocardial infarction, and non-fatal stroke compared to placebo [70,74,75]. In the case of exenatide and lixisenatide, the study showed no significant difference between the study group and placebo [73,76].

GLP-1 analogues show significant benefits in regulating glycemia and improving pancreatic β-cell function [77]. The results of many clinical trials confirm the efficacy of GLP-1 agonists in improving metabolic control, making them a substantial part of T2D therapy (Table 3). The clinical trials and pharmacological effects of GLP-1 analogues are summarized in Table 3.

#### 2.2.3. Pharmacokinetics of GLP-1 Analogues

In the development of GLP-1 analogues, changes in protein binding and prolongation of the half-life (t_1/2_) are of particular interest. The pharmacokinetics of GLP-1 analogues vary, affecting different dosing regimens and their clinical use. Formulations such as exenatide and lixisenatide, with shorter half-lives and higher clearance, require more frequent administration. In contrast, dulaglutide and semaglutide, with their long half-life and low clearance, can be administered weekly. Understanding the differences in how these drugs work makes it possible to tailor treatment to individual patients.

Immediate-release exenatide reaches T_max_ within 2 h. Its volume of distribution indicates a moderate spread of the drug in the body. Due to its short t_1/2_, frequent dosing is required to maintain therapeutic levels. Average clearance is 9 L/h, indicating relatively rapid elimination from the body [59,89].

Lixisenatide binds to proteins at a level of 55%. The volume of distribution is markedly higher than that of exenatide, indicating extensive distribution of the drug in the body. Clearance reaches 35 L/h, indicating relatively rapid excretion [58].

The bioavailability of dulaglutide varies with dose: for the 1.5 mg dose, it is 47%, while for the 0.75 mg dose, it is 65%. The volume of distribution is low, indicating limited distribution of the drug. The long t_1/2_ (5 days) and low clearance (0.142 L/h) indicate slow elimination from the body, allowing the drug to be administered once a week [61].

Liraglutide’s time to maximum plasma concentration and volume of distribution vary with the formulation, typically occurring after more than 8 h. The bioavailability of liraglutide is approximately 55%, while binding to plasma proteins exceeds 98%. Clearance (0.9–1.4 L/h) indicates a moderate rate of elimination from the body [57,90].

The bioavailability of semaglutide is exceptionally high at 89%. Binding to plasma proteins exceeds 99%, indicating high stability in this fluid. The half-life of semaglutide is longer than that of dulaglutide, at approximately 7 days, while its clearance rate (0.05 L/h) is comparatively lower. Slow metabolism and elimination allow the drug to be administered once a week [62,91].

The summary and details of the pharmacokinetic parameters of GLP-1 analogues are shown in Table 4.

#### 2.2.4. Summary of Clinical Trials Comparing GLP-1 Analogues with Other Classes of Compounds Used in T2D Therapy

Among oral hypoglycemic agents (OHAs), metformin is the first treatment for T2D. When metformin therapy fails, sulphonylureas and thiazolidinediones are commonly used. However, particularly sulphonylureas exert adverse effects such as significant weight gain and hypoglycemia. Furthermore, acarbose, which is sometimes co-administered with metformin, causes diarrhea, abdominal pain, and bloating. Despite the development of combination therapies of these drugs for managing diabetes, a large number of patients fail to achieve their blood glucose targets. Therefore, it seems that GLP-1 analogues are the leading drugs in T2D and metabolic syndrome therapy nowadays. The most significant effect of GLP-1 analogues compared to other OHAs is weight loss and a reduced risk of hypoglycemia [101].

Danish nationwide register data indicate that GLP-1RAs reduce the one-year risk of add-on glucose-lowering medication compared to metformin in prediabetes (risk ratio [RR]: 0.27, 95% confidence interval [CI]: 0.10–0.44) and diabetes (RR: 0.67, 95% CI: 0.37–0.98). Additionally, GLP-1 RA therapy leads to greater HbA_1c_ reduction compared to metformin (prediabetes: −2.59 mmol/mol, 95% CI: −3.10 to −2.09; diabetes: −3.79 mmol/mol, 95% CI: −5.28 to −2.30) [102]. Agonists of the GLP-1 receptor are a good option for obese patients with T2D who do not achieve adequate glycemic control with metformin monotherapy. Clinical evidence indicates that GLP-1 RA reduced BMI by 1.02 kg/m^2^ (95% CI: −1.46 to −0.58), fasting plasma glucose (FPG) by −21.34 mg/dL (95% CI: −29.53 to −13.15), and HbA_1c_ −0.56% (95% CI: −0.77 to −0.38) compared to other antidiabetic medications [103].

The GLP-1 analogue, such as liraglutide, used as add-on therapy to sulphonylureas, reduced HbA_1c_ by 1.0% (0.6 mg) and 1.27% (0.9 mg) compared to placebo. The effect of exenatide on lowering HbA_1c_ < 7% was significantly higher (observed in 42.2% of patients) than insulin glargine (in 21.0% of patients). It was reported that DPP-4 inhibitors less effectively control the reduction in HbA_1c_ than GLP-1 analogues, but they offer a lower risk of gastrointestinal discomfort [101]. It is noteworthy that liraglutide (0.9 mg/day), compared to glibenclamide (2.5 mg/day), significantly lowered FPG and postprandial glucose by the mean treatment difference −0.72 mmol/L and −5.2 mmol/L, respectively, at 24 weeks. However, liraglutide on FPG was effective after 4 weeks. In addition to the positive effects on glucose metabolism, the liraglutide effect on BNP (−10.71 pg/mL), plasminogen activator inhibitor-1 (PAI-1; −2.89 ng/mL), and hsCRP (−0.0886 mg/dL) was greater than that of glibenclamide. The lipid profile was only slightly improved by liraglutide [104].

The network meta-analysis and GRADE assessment showed that both SGLT-2 inhibitors and GLP-1 receptor agonists reduce all-cause mortality, cardiovascular mortality, non-fatal myocardial infarction, and kidney failure. SGLT-2 inhibitors are associated with a substantial reduction in the risk of heart failure hospitalization (odds ratio [OR] 0.70, 95% CI 0.63–0.77), while GLP-1 analogues demonstrate minimal to no impact in this regard (OR 0.94, CI 0.85–1.03). GLP-1 receptor agonists are more effective in reducing non-fatal stroke (OR 0.84, CI 0.76–0.93), while SGLT-2 inhibitors have minimal impact (OR 1.01, 95% CI 0.89–1.14). GLP-1 analogues reduce HbA1c levels to a greater extent than SGLT-2 inhibitors. There is high-certainty evidence that SGLT-2 inhibitors increase the risk of genital infections, while the association between GLP-1 analogues and serious gastrointestinal events is supported by low-certainty evidence [105,106].

Furthermore, another network meta-analysis demonstrated that SGLT-2 inhibitors, along with GLP-1RAs, had beneficial effects on mortality and major adverse cardiovascular events compared to DPP-4 inhibitors and glimepiride, particularly in high-risk populations, and were also the most effective in preventing hospitalization due to heart failure and kidney disease [107].

An important consideration is the comparison of medication non-adherence and non-persistence in individuals with T2D, given their substantial impact on treatment effectiveness and long-term disease control. The missed medication doses are described in the literature as adherence, compliance, or concordance, whereas persistence means duration of use before termination or substantial medication gap. Agonists of the GLP1 receptor, as administered subcutaneously, are differently tolerated by patients. Glucagon-like peptide-1 analogues demonstrated shorter persistence than DPP-4 inhibitors and lower adherence than sulphonylureas and DPP-4 inhibitors. To compare, long-acting insulin analogues had significantly longer persistence. GLP-1 analogue therapy was usually discontinued due to adverse effects, including injection site reactions and gastrointestinal disturbance [108].

### 2.3. Dipeptidyl Peptidase-4 Inhibitors

**Figure 3:**
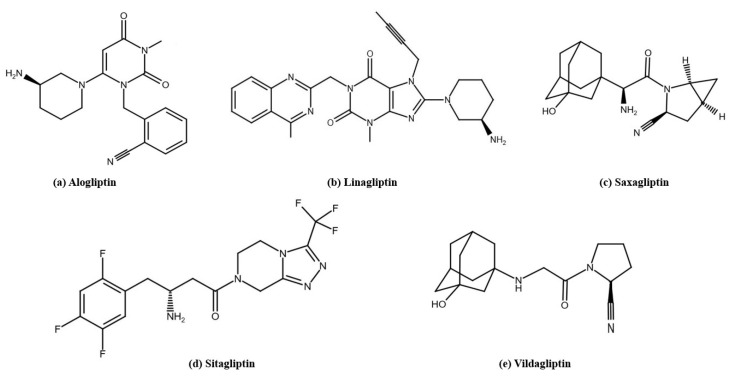
The structures of DPP-4 inhibitors used in T2D therapy: (**a**) alogliptin, (**b**) linagliptin, (**c**) saxagliptin, (**d**) sitagliptin, (**e**) vildagliptin. The structures were prepared with Reaxys.

Dipeptidyl peptidase-4 inhibitors (Figure 3), also known as gliptins, are prescribed to control blood sugar levels in adults with T2D. When combined with a healthy diet and regular exercise, these drugs can effectively help control blood sugar levels [109]. The mechanism of action of DPP-4 inhibitors involves inhibition of the breakdown of GIP and GLP-1. As a result, the action of incretin hormones is prolonged, promoting post-meal glucose regulation by increasing insulin secretion and reducing plasma glucagon levels [110]. Sitagliptin was the first DPP-4 inhibitor approved by the FDA in October 2006 (marketed as Januvia^®^, developed by Merck & Co., Inc. (Rahway, NJ, USA)) [111]. In March 2007, sitagliptin was also approved by the European Commission and marketed in all European Union [112]. The next drugs in this group approved by the FDA were saxagliptin in July 2009 (marketed as Onglyza^®^, developed by Bristol-Myers Squibb Company (New York, NJ, USA)) [113], linagliptin in May 2011 (marketed as Tradjenta^®^, developed by Boehringer Ingelheim Pharmaceuticals, Inc. (Ingelheim, Germany)) [114], and alogliptin in January 2013 (marketed as Nesina^®^, developed by Takeda Pharmaceutical Company (Cambridge, MA, USA)) [115]. In addition, vildagliptin was approved in the European Union in September 2007 (marketed as Galvus^®^, developed by Novartis Europharm Limited (London, United Kingdom)) [116,117]. Gliptins are available as stand-alone drugs and in combination with other diabetes medications, such as metformin [109].

#### 2.3.1. Drug Manufacturing

The synthesis of saxagliptin (BMS-477118) started with the preparation of vinyl fragments by Horner–Emmons olefination reaction and Claisen rearrangement. The resulting vinyl amino acids were coupled to L-cis-4,5- methanoprolinamide under standard peptide coupling conditions using hydroxybenzotriazole and [1-ethyl-3-(3-dimethylaminopropyl) carbodiimide hydrochloride] (HOBT/EDC) in dimethylformamide. The products were dehydrated to nitriles using phosphoryl chloride (POCl_3_), and the bioactive L isomer was isolated chromatographically and subjected to TFA deprotection. In further steps, the vinyl side groups were functionalized, including ozonolysis, and the introduced adamantylglycine fragment was obtained by Strecker reaction. This method of synthesis provided saxagliptin with high activity against DPP-4 and a favorable pharmacokinetic profile [118].

The synthesis of sitagliptin is based on several key steps. The process begins with the synthesis of triazolopiperazine, which is formed by the reaction of chlorpyrazine with hydrazine. The key step is the conversion of α-amino acid to β-amino acid by Arndt–Eistert homologation. The β-amino acid is then coupled to triazolopiperazine in a standard peptide reaction, and the final step is the removal of the Boc protecting group, resulting in pure sitagliptin. On an industrial scale, a key innovation was the asymmetric reduction in enamines using a rhodium-based catalyst and Joshiphos ligand, which simplified the synthesis, eliminating the need for protecting groups and increasing the efficiency of the process [119].

The synthesis process of alogliptin is based on the structure of pyrimidinedione, the chemical basis of the compound. The steps include selective alkylation, methylation, and a reaction to replace the chlorine atom with a 3-(R)-aminopiperidine group to produce the final alogliptin molecule, known as SYR-322 [120].

The synthesis of vildagliptin involves three steps. It begins with the acylation of L-proline amide with chloroacetyl chloride, resulting in an intermediate compound. This admixture then undergoes dehydration with Vilsmeier’s reagent produce cyanopyrrolidine. The final step is the alkylation of cyanpyrrolidine with hydroxyaminoadamantane in the presence of a base, resulting in vildagliptin [117].

The synthesis of linagliptin is based on the optimization of a compound based on the xanthine backbone. Key steps involve the selective introduction of substituents at the N-1, N-7, and C-8 positions via alkylation and halogen exchange reactions. The highest biological activity is achieved with a but-2-ynyl substituent at the N-7 position and a 3-aminopiperidine group at the C-8 position, which provides strong binding to the DPP-4 enzyme. These modifications produce a highly selective inhibitor with long-lasting activity [121].

#### 2.3.2. Mechanism of Action and Pharmacological Effects

The mechanism of action of DPP-4 inhibitors is to prolong the activity of incretins, such as GLP-1 and GIP, by inhibiting their degradation (Figure 2). The drugs help regulate postprandial blood sugar levels, primarily by reducing glucagon levels and enhancing insulin secretion by the pancreas [81,110]. DPP-4 inhibitors also contribute to lowering fasting blood glucose levels [110].

The clinical trials and pharmacological effects of DPP-4 inhibitors are summarized in Table 3.

#### 2.3.3. Pharmacokinetics of DPP-4 Inhibitors

The pharmacokinetics of DPP-4 inhibitors are characterized by variability in key parameters such as T_max_, bioavailability, binding to plasma proteins, volume of distribution, and renal clearance (Table 4). These differences affect the pharmacological activity of individual drugs and their clinical use.

Alogliptin has a bioavailability of 100%, indicating complete absorption of the drug. The binding of alogliptin to plasma proteins is relatively low (20–30%). Renal clearance is 170 mL/min, and the half-life is as high as 21 h (Table 4), allowing the drug to be used once daily [92].

Linagliptin has a bioavailability of 30%. The binding of the drug to plasma proteins is concentration-dependent: it is 99% at a concentration of 1 nmol/L and 75–89% at a concentration ≥ 30 nmol/L. The mean apparent volume of distribution after intravenous administration (Table 4) indicates significant distribution in tissues. Renal clearance at steady state is approximately 70 mL/min [93].

The mean AUC of saxagliptin was determined to be 78 ng·h/mL. Saxagliptin shows negligible binding to plasma proteins. Renal clearance is approximately 230 mL/min. Its short half-life means that its active metabolite plays an important role in prolonging the effect of the drug [94].

The mean plasma AUC for sitagliptin is 8.52 µM·h/mL and the bioavailability of the drug is high at 87%. The medication is 38% bound to plasma proteins. Renal clearance is approximately 350 mL/min [95].

Vildagliptin has a bioavailability of 85% and low binding to plasma proteins (9.3%). Intravenous plasma clearance is 4 L/h, and renal clearance is 13 L/h. The half-life of the drug depends on the route of administration (Table 4) [96].

In summary, the pharmacokinetic profiles of DPP-4 inhibitors differ, which may influence their therapeutic efficacy and guide the selection of the most appropriate agent for individual patients.

#### 2.3.4. Summary of Clinical Trials Comparing Dpp4 Inhibitors with Other Classes of Compounds Used in T2d Therapy

In a comparative effectiveness study, DPP-4 inhibitors and sulphonylureas were less effective than SGLT-2 inhibitors in reducing HbA_1c_ levels, BMI, and systolic blood pressure. On the other hand, compared to DPP-4 inhibitors and sulphonylureas, SGLT-2 inhibitors showed more advantages in preventing hospital admissions for heart failure and ≥40% decline in eGFR [122].

Additionally, a review of randomized controlled trials (1974–2012 years) of DPP-4 inhibitors and metformin found that DPP-4 inhibitors had smaller effect on HbA_1c_ (MD = 0.28), FPG (MD = 0.81), and weight loss (MD = 1.51) but were more effective in reducing the risk of cardiovascular events (RR = 0.36), hypoglycemia (RR = 0.44), and gastrointestinal side effects (RR = 0.63). Combining DPP-4 inhibitors with metformin resulted in greater reductions in HbA_1c_ (MD = −0.49) and FPG (MD = −0.80) but did not further reduce cardiovascular events (RR = 0.54), hypoglycemia (RR = 1.04), or gastrointestinal side effects (RR = 0.98) [123]. In cases of mild, moderate, or severe hypoglycemia, DPP-4 inhibitors were preferred over sulphonylureas due to their more favorable safety profile. On the other hand, metformin was favored more than DPP-4 inhibitors based on the comparison of monotherapies in the field of differences in the change in HbA_1c_ and weight [124].

In the meta-analysis by McGovern et al., DPP-4 inhibitors demonstrated superior patient adherence and longer treatment persistence compared to sulphonylureas and thiazolidinediones. One study reported that α-glucosidase inhibitors and meglitinides were associated with lower adherence rates compared to other OHAs. Additionally, canagliflozin demonstrated greater treatment persistence than DPP-4 inhibitors in the same analysis [108].

### 2.4. Sodium Glucose Transporter-2 Inhibitors

**Figure 4:**
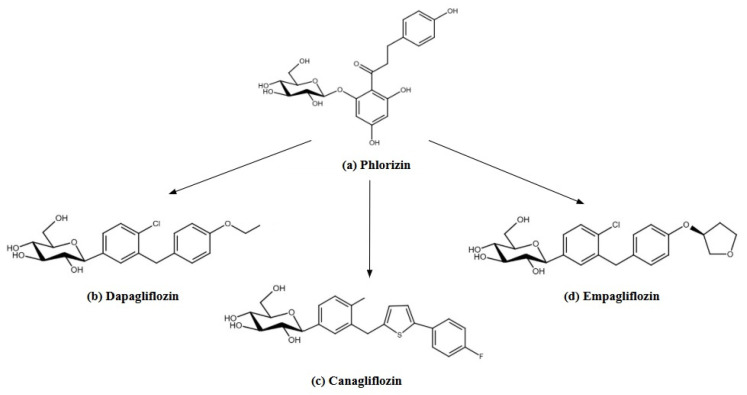
The structures of phlorizin (**a**) as a precursor of SGLT-2 inhibitors: (**b**) dapagliflozin, (**c**) canagliflozin, (**d**) empagliflozin. The structures were prepared with Reaxys.

Sodium glucose transporter-2 inhibitors are a new group of drugs used in the treatment of T2D. Their action is to block glucose reabsorption in the kidneys, which leads to glucosuria and results in lower blood glucose levels. This mechanism works independently of insulin, making these drugs suitable for use as monotherapy and in combination with other antidiabetic agents, such as metformin or insulin [125]. The precursor of SGLT2 inhibitors was phlorizin (phloretin 2-glucoside), a natural compound isolated from the bark of apple trees in 1835. Phlorizin (Figure 4) has a bitter taste that contributes to the characteristic flavor of cider, while its dimerized oxidation products contribute to the color of apple juices. In the 1990s and 2000s, its therapeutic potential was recognized, following the development of selective and stable synthetic inhibitors. The first FDA-approved SGLT2 inhibitor, canagliflozin (marketed as Invokana^®^, developed by Mitsubishi Tanabe Pharma (Osaka, Japan), was introduced in March 2013, marking a significant advancement in diabetes and cardiovascular care. Extensive evidence from industry-sponsored clinical trials supports the safety and efficacy of SGLT2 inhibitors in enhancing glycemic control in adults with T2D. Approval of canagliflozin was followed by the approval of dapagliflozin (marketed as Farxiga^®^, developed by AstraZeneca (Cambridge, United Kingdom) and Bristol-Myers Squibb Company (New York, NJ, USA)) in January 2014 and empagliflozin (marketed as Jardiance^®^, developed by Boehringer Ingelheim (Ingelheim, Germany)) in August 2014 [126].

Currently, the FDA and EMA have approved three drugs within this class of compounds [125]. New substances such as ipragliflozin, tofogliflozin, and sotagliflozin are also in clinical trials, which may further expand the use of this class of drugs in the future [127]. Although SGLT-2 inhibitors have shown high efficacy in the treatment of T2D, their use in T1D has not been approved by the FDA due to insufficient data on safety and efficacy [125].

#### 2.4.1. Drug Manufacturing

Phlorizin, despite its promising therapeutic effects, has drawbacks that limit its clinical use [126]. Intestinal enzymes quickly degrade it into its constituent molecules, glucose and phloretin. Low bioavailability of phlorizin limits the amount of active ingredient reaching the body. In addition, the use of phlorizin orally leads to osmotic diarrhea, as it blocks glucose absorption in the intestines, a serious side effect that hinders its practical use. Additionally, a metabolite of phlorizin non-specifically inhibits glucose transporters (GLUTs). This, in turn, can interfere with glucose uptake by various tissues, potentially affecting metabolism throughout the body [128].

Given the various shortcomings of phlorizin, pharmaceutical companies considered developing more stable and selective therapeutic agents. The initial focus was on *O*-glucosides [128]. In 1999, Oku et al. presented research on the newly synthesized compound T-1095 and its active metabolite T-1095A [129]. Katsuno et al., and Fujimori et al., published studies on sergliflozin and remogliflozin, respectively. Both drugs were manufactured by Kissei Pharmaceutical Co., Ltd. (Azumino, Nagano, Japan) [130,131]. In 2009, Bickel et al. presented the drug AVE2268, synthesized by the Sanofi-Aventis Deutschland GmbH, Frankfurt/Main, Germany [132]. Despite confirmed efficacy and well-characterized effects in humans, the pharmacokinetic limitations of these compounds appear to have impeded their clinical development [133]. Taking this into account, research on *C*-glucosides has begun.

Dapagliflozin was developed as an effective and selective blood-glucose-lowering drug. Its unique structure is based on a *C*-glucosidic bond, which makes it more resistant to enzymatic degradation compared to *O*-glucosidic compounds. Dapagliflozin is resistant to hydrolysis by intestinal, hepatic, and renal glucosidases, increasing its stability and efficacy [134]. Persilylated gluconolactone was synthesized by reacting with trimethylsilyl chloride. Friedel–Crafts acylation of phenetole with 5-bromo-2-chlorobenzoyl chloride produced *p*-benzophenone, which was reduced to the aglycone. Lithium–halogen exchange with persilylated gluconolactone formed lactols, converted to *O*-methyl lactols with methanesulfonic acid. Final reduction, peracetylation, and hydrolysis yielded dapagliflozin [134].

Canagliflozin is produced in several steps, using *C*-glucoside derivatives with a heteroaromatic ring, such as thiophene. The process begins with the synthesis of aglycones, which are formed by Friedel–Crafts acylation using benzol chloride and thiophene, followed by ketone reduction using triethylsilane and boron trifluoride [135]. Aglycones are dissolved in tetrahydrofuran and toluene, treated with *n*-butyllithium at −78 °C, resulting in aryllithium. 2,3,4,6-*tetra*-*O*-trimethylsilyl-*β*-D-gluconolactone is added to the ensuing mixture, indirectly in anomeric lactosides. The lactosides are converted to methyl ethers using methanesulfonic acid in methanol. In summary, *C*-glucoside derivatives are obtained by stereoselective reduction using triethylsilane and boron trifluoride in dichloromethane [135]. In optimizing the structure of canagliflozin, special attention is paid to the selection of appropriate substituents in the aromatic ring [135]. High selectivity toward the SGLT2 co-transporter and chemical stability make canagliflozin an effective drug for T2D.

Wang et al., developed a modern and efficient method for the synthesis of empagliflozin. Their research focused on optimizing a chemical process amenable to industrial-scale production, with an emphasis on maintaining high product yields and purity. The entire procedure consists of four main chemical steps. The initial step involves an I/Mg exchange reaction of aryl iodide. This admixture then reacts with gluconolactone, leading to the formation of β-anomeric methyl glycopyranoside. The intermediate thus produced is subjected to further reactions, eliminating the need for its isolation [136]. The most important step in the synthesis is reduction using Et3SiH in the presence of aluminum chloride (AlCl_3_), which acts as an acid catalyst. This process is distinguished by its high selectivity toward β-*C*-glycoside, a key structural element of empagliflozin [136].

#### 2.4.2. Mechanism of Action and Pharmacological Effects

Sodium-glucose transporters are responsible for reabsorbing ~90% of filtered glucose. SGLTs-2 are also present in the heart, brain, muscle, liver, thyroid, and pancreatic α-cells [126,137].

Clinical trials have demonstrated that SGLT2 inhibitors confer unexpected benefits beyond glycemic control, including a 38% reduction in the risk of major cardiovascular events, delayed progression of chronic kidney disease, a 35% decrease in hospitalization rates for heart failure, and a lower incidence of acute kidney injury [138,139]. These findings have broadened the therapeutic applications of SGLT2 inhibitors beyond T2D, supporting their use in the management of cardiovascular and renal diseases [139]. The cardioprotective benefits of SGLT-2 inhibitors result from a multifaceted action, including improving cardiac function, reducing the risk of arrhythmias, and modifying myocardial energy metabolism [140]. According to the EMPA-REG OUTCOME trial, SGLT-2 inhibitors increase the production of ketone bodies, which support the mitochondria of cardiomyocytes, and potentiate ATP production, leading to an increase in cardiac contractility [138]. Changes in intracellular sodium and calcium lead to improved cardiac contractile function and a reduced risk of arrhythmias [141]. Furthermore, a reduction in epicardial adipose tissue (EAT) promotes improvement of heart health. A decrease in glucose uptake is associated with a reduction in resting myocardial blood flow and an improvement in the reserve of this circulation. It is hypothesized that SGLT-2 inhibitor therapy restores the anti-inflammatory properties of EAT, leading to enhanced coronary microvessel function. Through these actions, drugs help improve cardiac function and reduce cardiovascular burden [85]. According to a meta-analysis performed by Baker et al., SGLT-2 inhibitors cause a sustained reduction in systolic blood pressure by about 4.34 mmHg and diastolic blood pressure by about 2.61 mmHg [142]. These effects are likely due to several mechanisms, including plasma volume reduction by osmotic diuresis, weight reduction, improved vascular elasticity, and reduced oxidative stress associated with hyperglycemia. In addition, SGLT-2 inhibitors can reduce sympathetic nervous system activity [127].

Inhibitors of SGLT2 show significant nephroprotective effects, especially in patients with T2D and chronic kidney disease (CKD). By increasing sodium chloride delivery to the macula densa, these drugs regulate tubulointerstitial coupling. They reduce glomerular hyperfiltration and lower intraglomerular pressure [143].

Inhibitors of SGLT2 have shown promise in improving non-alcoholic fatty liver disease (NAFLD). Studies including a meta-analysis by Mantovani et al., have shown that the use of SGLT2 inhibitors, such as empagliflozin, dapagliflozin, canagliflozin, and ipragliflozin, is associated with a significant reduction in liver fat content, as assessed by magnetic resonance imaging [144]. Reductions in liver enzymes such as alanine aminotransferase and *γ*-glutamyltransferase, which are markers of liver damage, were also observed [144]. This evidence suggests a beneficial effect of this class of drugs on hepatic steatosis, particularly in patients with T2D. However, further studies are needed to confirm their efficacy in patients without glycemic disturbances.

Inhibitors of SGLT-2 exert modest effects on the lipid profile, including a reduction in triglyceride levels and an increase in both HDL and LDL cholesterol concentrations [145].

The clinical trials and pharmacological effects of SGLT-2 inhibitors are summarized in Table 3.

#### 2.4.3. Pharmacokinetics of SGLT-2 Inhibitors

Canagliflozin is characterized by rapid absorption, reaching maximum plasma concentration within 1–2 h [97]. The area under the plasma drug concentration-time curve (AUC∞) for the 100 mg dose is 6.818 ng·h/mL, and for the 300 mg dose it is as high as 22.953 ng·h/mL, reflecting a marked increase in drug exposure with increasing dose [98]. The bioavailability of canagliflozin is 65% [97]. The drug binds to plasma proteins to a very high degree (99%) and distributes in an average volume (Table 4). Canagliflozin is metabolized by the UGT1A9 and UGT2B4 enzymes. The mean total systemic clearance of canagliflozin is 192 mL/min [97].

Dapagliflozin 10 mg reaches maximum plasma concentration fairly rapidly (Table 4) with a bioavailability of 78%. The drug binds to plasma proteins in 91%. It is mainly metabolized by the uridine 5′-diphospho-glucuronosyltransferase (UGT)1A9. Total systemic clearance is 207 mL/min [99].

Empagliflozin is characterized by rapid absorption (Table 4). For a dose of 10 mg, the C_max_ is 259 nmol/L, rising to 687 nmol/L for a dose of 25 mg. The area under the plasma drug concentration–time curve for the 10 mg dose is 1.870 nmol·h/L, and for the 25 mg dose it increases to 4.740 nmol·h/L, showing a marked increase in drug exposure with increasing dose [100]. Empagliflozin is 86% bound to plasma proteins. The drug is metabolized by enzymes such as UGT2B7, UGT1A3, UGT1A8, and UGT1A9. Apparent systemic clearance is estimated at 10.6 L/h, indicating relatively slow elimination of the drug from the body [100].

Although dapagliflozin, canagliflozin, and empagliflozin differ in their pharmacokinetic profiles, they all exhibit rapid absorption and a long half-life, enabling once-daily dosing.

#### 2.4.4. Summary of Clinical Trials Comparing SGLT-2 Inhibitors with Other Classes of Compounds Used in T2D Therapy

Nationwide cohort data show no significant differences between SGLT2 inhibitor-based and metformin-based regimens in patients with diabetes and low cardiovascular risk (mean age 62.0 ± 11.6 years, 50% male). However, in patients under 65 years, SGLT2 inhibitors were associated with lower all-cause mortality (HR: 0.47, 95% CI: 0.23–0.99) and reduced end-stage renal disease (HR: 0.22, 95% CI: 0.06–0.82) [146]. In a network meta-analysis of nine studies including 87,162 participants, SGLT-2 inhibitors were found to reduce the risk of heart failure hospitalization significantly compared to placebo (RR: 0.56; 95% CI: 0.43 to 0.72), as well as in pairwise comparisons with GLP-1 agonists (RR: 0.59; 95% CI: 0.43 to 0.79) and DPP-4 inhibitors (RR: 0.50; 95% CI: 0.36 to 0.70) [147]. Inhibitors of SGLT-2 showed statistically significant reduction in mortality compared to DPP-4 inhibitors (−1.15; −1.76, −0.47; *p* < 0.05) and the sulphonylureas (−1.88; −2.94, −0.62; *p* < 0.05) represented by glimepiride. In addition, when SGLT-2 inhibitors were compared to sulphonylureas, they more efficiently reduced the major adverse cardiovascular event (−1.59; −3.17, 0.23) and hospitalization for heart failure (−0.64; −1.25, 0.18) [107]. In the context of chronic kidney disease and diabetes, available data comparing SGLT2 inhibitors with standard care, sulphonylureas, DPP-4 inhibitors, or insulin remain inconclusive and are characterized by a degree of uncertainty [148]. There is low to moderate evidence that SGLT2 inhibitors and GLP1 agonists reduce severe hypoglycemia in comparison with sulphonylureas and insulin [149]. The comparison of SGLT-2 and metformin indicated that in the cases of genital mycotic infections, metformin is favored [124].

Some reports showed that SGLT-2 inhibitors appeared to lower body weight [−1.92 kg (95% CI −2.23 to −1.62)] to a greater extent than GLP-1 receptor agonists [−0.47 kg (−0.85 to −0.09)] from low to moderate certainty [105]. SGLT2 inhibitors were significantly associated with lower incidence rates of adverse liver-related outcomes [adjusted subdistribution hazard ratio (ASHR), 0.37, 95% CI, 0.17–0.82] and non-alcoholic fatty liver disease (ASHR, 1.99, 95% CI, 1.75–2.27) when compared to sulphonylureas [150].

## 3. Antilipemic Drugs

### 3.1. PCSK9 Inhibitors

The history of the discovery of PCSK9 inhibitors begins with the identification of the PCSK9 gene, which is located on chromosome 1 (locus: 1p33–34.3), and its effect on cholesterol metabolism. In 2003, Abifadel et al. established that mutations within this gene are responsible for autosomal dominant hypercholesterolemia (ADH) [151]. Similar conclusions were also reached by Leren et al., who conducted a study on 51 patients with familial hypercholesterolemia [152].

In 2015, alirocumab (marketed as Praluent^®^, developed by Sanofi Winthrop Industrie (Gentilly, France)), as the first PCSK9 inhibitor, received approval from the FDA and subsequently also from the EMA [153,154]. Another drug in this group is evolocumab (marketed as Repatha^®^, developed by Amgen Europe B.V. (Breda, The Netherlands)), which was also launched in 2015 [155,156]. The proprotein convertase subtilisin/kexin type 9 has combined indications for the treatment of children and adults. Alirocumab is indicated in children over 8 years of age, while evolocumab is indicated in children over 10 years of age. These drugs are approved for the treatment of heterozygous hypercholesterolemia, both familial and non-familial, and mixed dyslipidemia. Another indication for PCSK9 inhibitors is diagnosed myopic cardiovascular disease. Alirocumab and evolocumab are used in combination therapy alongside a statin or in conjunction with a statin paired with another hypolipemic drug. If statins are intolerable or ineffective, PCSK9 inhibitors can be used in monotherapy or combination therapy with other hypolipemic drugs. Evolocumab is also indicated for the treatment of homozygous familial hypercholesterolemia in combination with other hypolipemic medications [153,156].

#### 3.1.1. Drug Manufacturing

Alirocumab and evolocumab are human monoclonal antibodies, IgG1 and IgG2, respectively, administered subcutaneously [157]. The synthesis of both drugs is based on recombinant DNA technology. The genes encoding the light and heavy chains of the antibodies are cloned into expression vectors containing the hCMV-MIE promoter. They are then introduced into Chinese hamster ovary cells, where they are expressed [157,158].

#### 3.1.2. Mechanism of Action and Pharmacological Effects

The proprotein convertase subtilisin/kexin type 9 is an enzyme belonging to the serine endoprotease family. The main site of PCSK9 synthesis is the liver, and to a lesser extent the intestines, kidneys, and brain. The mechanism of action of this protein is to bind to and degrade LDL receptors, which leads to an increase in blood LDL levels. By mapping the interaction between PCSK9 and LDLR, it has become possible to develop drugs that inhibit the action of this enzyme [159]. The mode of action of alirocumab and evolocumab is to bind to circulating PCSK9, preventing its interaction with LDL receptors and allowing LDLR to return to the surface of liver cells. This increases the number of LDL-receptors ready to degrade low-density lipoproteins. As a result, LDL-C levels in the blood are significantly reduced [155].

#### 3.1.3. Pharmacokinetics of PCSK9 Inhibitors

Evolocumab reaches a maximum concentration of 13 μg/mL at dose 140 mg and 46 μg/mL at dose 420 mg within 3–4 days, whereas the time to peak concentration of alirocumab is slightly longer, ranging from 3 to 7 days [153,156]. Evolocumab has a moderate volume of distribution of approximately 3.3 L [156]. Both drugs have a relatively long half-life, from 17 to 20 days for alirocumab (doses of 75 mg and 150 mg) and from 11 to 17 days for evolocumab, reflecting their slow elimination from the body [153,156].

The randomized controlled FOURIER trial included 25,096 patients with established atherosclerotic cardiovascular disease. After 48 weeks of evolocumab, LDL-cholesterol and Lp(a) levels were examined. It was shown that evolocumab reduced LDL-C concentration by 59% from a median baseline value and Lp(a) by a median (interquartile range) of 26.9% (6.2–46.7%). This drug has also been shown to decrease the risk of mortality due to myocardial infarction, coronary heart disease, or the necessity for urgent revascularization by 23% [160].

The ODYSSEY OUTCOMES, a multicenter, double-blind trial, involved 18,924 patients with recent acute coronary syndrome, randomly assigned to receive alirocumab subcutaneously at a dose of 75 mg or matching placebo every 2 weeks. Alirocumab significantly lowered LDL-C (median −51.3 [−67.1, −34.0] mg/dL) and Lp(a) (median −5.0 [−13.6, 0] mg/dL) and the risk of cardiovascular events. Alirocumab led to a better therapeutic effect in patients with initially higher Lp(a) levels. Specifically, each 5 mg/dL decrease in Lp(a) was associated with a 2.5% relative reduction in the occurrence of cardiovascular incidents [161].

#### 3.1.4. Summary of Clinical Trials Comparing PCSK9 Inhibitors and Statins

Inhibitors of PCSK9 are particularly suitable for patients whose cholesterol levels cannot be effectively controlled by statins or other conventional treatments. The reported limitations include higher treatment costs and adverse effects such as injection site reactions, influenza-like symptoms, and upper respiratory tract infection-related manifestations [162]. However, PCSK9 inhibitors are highly effective in improving lipid profiles. In a meta-analysis of 84 randomized controlled trials involving 246,706 patients, they were most effective in enhancing lipid outcomes. For example, PCSK9 inhibitors were found to be more effective than statins in reducing LDL cholesterol (−16.74, 95% CI −28.87 to −4.60) and total cholesterol (TC; −11.06, 95% CI −16.21 to −5.91), and increasing HDL cholesterol (3.20, 95% CI 1.40 to 4.99). Statins were slightly better for reducing cardiovascular events, but with no significant difference from PCSK9 inhibitors. Statins were not superior to PCSK9 inhibitors for improving all-cause mortality. In addition, they increased alanine aminotransferase (ALT; OR 1.89, 95% CI 1.42–2.51), creatinine kinase (OR 1.45, 95% CI 1.09–1.93), and diabetes risk (OR 1.13, 95% CI 1.02–1.26), whereas PCSK9 inhibitors had no significant adverse effects [163]. In addition to these findings, a review of 33 studies (23,375 patients) comparing three treatments on lipid levels revealed that PCSK9 inhibitors reduced LDL-C more than statins (−11.36, 95% CI: −24.16 to 1.43). The combination of statins and ezetimibe achieved statistical significance over PCSK9 inhibitors in lowering triglyceride (TG) levels. It is worth noting that PCSK9 inhibitors guarantee short-term rapid LDL-C reduction, while inclisiran, a siRNA that prevents the liver from producing the PCSK9 protein, focuses more on long-term maintenance of low LDL-C levels. The safety outcomes for different classes of compounds can be summarized as follows: PCSK9 inhibitor with ezetimibe > statin with ezetimibe > statin > inclisiran > PCSK9 inhibitors > ezetimibe [162].

### 3.2. Antisense Oligonucleotides

In 1978, Stephenson and Zamecnik demonstrated that tridecamer oligodeoxynucleotides can effectively block viral replication [164]. The first approved drug from this group of compounds was fomivirsen. This medication was marketed for the treatment of patients diagnosed with cytomegalovirus retinitis [165]. The first ASO used in the management of homozygous familial hypercholesterolemia was mipomersen (marketed as Kyanmro^®^_,_ developed by Genzyme Europe BV (Amsterdam, The Netherlands)), an antisense oligonucleotide inhibitor of apolipoprotein B-100 (apoB-100). However, mipomersen has adverse effects on the liver, and mechanisms of liver damage other than steatosis cannot be ruled out. Notably, steatosis is likely to correlate with the degree of cholesterol reduction, raising additional concerns about long-term use of this medicine. A three-fold increase in serum ALT and aspartate transaminase (AST) levels above the upper limit of normal, and/or the presence of liver toxicity, is considered clinically significant. For this reason, it is no longer commercially available in the U.S. [166].

To date, no ASO has been approved for the treatment of hypercholesterolemia. However, a promising ASO-drug is in clinical trials. Pelacarsen, a potent inhibitor of Lp(a) synthesis, is currently in phase 3 of the HORIZON clinical trial (NCT04023552) [167]. This study aims to elevate the potential indication of this drug in the context of cardiovascular risk reduction in patients with cardiovascular disease and elevated Lp(a) levels [167]. The trial started on 12 December 2019. The anticipated completion is scheduled for February 2026. However, an antisense oligonucleotide, called volanesorsen, was approved in 2019 by the EMA for use in adult patients diagnosed with familial chylomicronemia syndrome [168]. Volanesorsen is an ASO that selectively binds to apoC-III messenger RNA, inhibiting its translation and promoting its degradation via RNase H-mediated cleavage. This mechanism results in a marked reduction in circulating apoC-III levels and a corresponding decrease in plasma TG concentrations [168].

#### 3.2.1. Drug Manufacturing

Pelacarsen is a second-generation 2′-methoxyethyl chimeric ASO that has been covalently linked to triantennary N-acetylgalactosamine (GalNAc), which provides uptake by hepatocytes via the asialoglycoprotein receptor [169,170].

Volanesorsen is a second-generation ASO targeting human APOC3 mRNA. The drug is a 20-mer oligonucleotide, with its nucleotides linked by phosphorothioate bonds. The structure of volanesorsen consists of three regions. Specifically, at the 5′ and 3′ ends, there are five nucleotides each with modified 2′-*O*-(2-methoxyethyl) ribonucleotides, while the middle part is made up of ten oligodeoxynucleotides [171].

Transformations of ASOs primarily involve altering the chemical structure of selected nucleotides, including modifications of sugar residues and modifications of phosphodiester bonds. Such structural modifications prevent premature degradation of the molecule and, in addition, can increase the efficiency and selectivity of oligonucleotides for complementary sequences. The most prevalent modifications employed in ASO are alterations at the 2′-ribose position. These transformations encompass 2′-*O*-methyl, 2′-*O*-methoxyethyl, locked nucleic acids, and also 2′-fluoro RNA. The introduction of such modifications is compatible with DNA and RNA synthesis methods. As a result, it is possible to fine-tune the properties of oligonucleotides to specific targets [172].

#### 3.2.2. Mechanism of Action and Pharmacological Effects

Antisense oligonucleotides are short, single-stranded nucleotide sequences designed to bind to the target RNA through complementary sequences using the Watson–Crick base pairing. Following subcutaneous administration, ASOs enter cells and localize to the nucleus, where they bind to target mRNA, activate RNase H, and ultimately induce mRNA degradation [173]. The hybridization of ASO to the RNA effectively blocks apo(a) expression. Furthermore, the antisense strand shows resistance to cleavage and can bind to additional target mRNA, leading to an extended half-life of these biologics [169].

Apolipoprotein C-III (apoC-III) is considered to be a key regulator of lipoprotein metabolism, particularly of TG. A component of TG-rich lipoproteins, its action is to inhibit lipoprotein lipase and retard TG clearance, leading to hypertriglyceridemia. Volanesorsen’s mechanism of action involves hybridization with cognate mRNA, leading to degradation of APOC3 mRNA via RNase H1 activity. As a result, this prevents the synthesis of apoC-III [171].

#### 3.2.3. Pharmacokinetics of ASO

##### Pelacarsen

Participants in the phase 1/2a clinical trials of pelacarsen were healthy volunteers with an Lp(a) level of at least 75 nmol/L who were randomly assigned to different dosing regimens. The single-dose part (10–120 mg) used a 3:1 dose escalation schedule versus placebo, while the multiple-dose part (10 mg, 20 mg, and 40 mg) gave participants the drug on days 1, 3, 5, 8, 15, and 22 (8:2 ratio versus placebo). The 2015–2016 study included 58 participants. In the groups receiving multiple doses of pelacarsen, there was a significant reduction in Lp(a) levels: by 66% at the 10 mg dose, 80% at the 20 mg dose, and 92% at the 40 mg dose. Lipoprotein(a) levels were also markedly reduced in patients taking a single dose of pelacarsen [170]. The randomized, double-blind phase 2 study involved 286 patients with cardiovascular disease and with Lp(a) levels above 60 mg/dL. Trial participants received pelacarsen at doses of 20 mg, 40 mg, and 60 mg at different intervals or a placebo. The slightest decrease in Lp(a) concentration was observed with the 20 mg dose administered monthly, while the greatest decrease was recorded with the 60 mg dose delivered monthly and with the 20 mg dose administered weekly, by 72% and 80%, respectively. For patients receiving placebo, Lp(a) levels reduced by only 6% [169].

In addition, the results of clinical studies indicate that pelacarsen also affects the reduction in apo B, LDL-C, and oxidized phospholipids, which are associated with apolipoproteins [169,170].

##### Volanesorsen

Volanesorsen, administered at 285 mg once a week, reaches a maximum concentration of 8.92 μg/mL within 2–4 h of subcutaneous administration. The medication has a large estimated volume of distribution at steady state (V_ss_) in patients with familial hypertriglyceridemia of up to 330 L. This ASO has a strong affinity for human plasma proteins, exceeding 98%. The half-life of volanesorsen is approximately 2–5 weeks [168].

The randomized double-blind phase 3 COMPASS study included 114 participants with multifactorial severe hypertriglyceridemia or familial chylomicronemia syndrome with TG levels above 500 mg/dL. Each patient had to be at least 18 years of age and have a BMI of 45 kg/m^2^ or less. Participants were randomly divided into groups receiving 300 mg of volanesorsen or placebo. The COMPASS study demonstrated that taking volanesorsen for three months reduced TG concentration by 71.2% while there was only a 0.9% decrease when taking a placebo. Volanesorsen may also contribute to reducing the incidence of acute pancreatitis in patients with multifactorial chylomicronemia [174].

The APPROACH, randomized double-blind phase 3 trial involved 66 participants with known familial chylomicronemia syndrome. Patients were randomly divided into groups receiving volanesorsen or placebo. The APPROACH study showed that the use of volanesorsen for three months resulted in a mean decrease in plasma apoC-III levels of 84%, while a mean increase in apoC-III concentration of 61% was observed in patients receiving placebo. Triglyceride levels examined after three months of therapy were reduced by up to 77%. For patients in the placebo group, a mean increase of 18% in TG concentration was observed [175].

#### 3.2.4. Summary of Clinical Trials Comparing ASO Drugs with Other Classes of Lipid-Lowering Drugs

Due to preliminary clinical trials, the majority of ASO were implemented in hypercholesterolemic subjects receiving stable statin therapy. Mipomersen on top of statin therapy resulted in an increased proportion of patients reaching LDL-target levels [176]. On the other hand, mipomersen was tested in statin-intolerant subjects at high risk for cardiovascular disease. The drug at 200 mg/week resulted in a significant reduction in LDL of 47% [177]. To our knowledge, no study has directly compared the lipid-lowering efficacy of ASOs with other classes of drugs. It can be concluded that, apart from the withdrawn mipomersen, other ASOs are at an early stage of development.

### 3.3. Small Interfering RNA Drugs

For the past two decades, therapies based on siRNA have been developed [178]. The first approved drug in this group for the treatment of heterozygous familial or non-familial hypercholesterolemia and mixed dyslipidemia is inclisiran (marketed as Leqvio^®^, developed by Novartis Europharm Limited (London, United Kingdom)). In December 2020, inclisiran was approved by the EMA and launched on the European market [179]. After one year, in 2021, the medication also received approval from the FDA and was indicated for clinical atherosclerotic cardiovascular disease, where LDL-C reduction is required [178,180]. Several other drugs in this class are currently in clinical trials for the treatment of hypercholesterolemia. One of these medications is olpasiran, which is currently in phase 3 of the OCEAN(a) clinical study (NCT05581303) [181]. This study aims to evaluate the effectiveness of olpasiran in reducing the risk of cardiovascular death in patients with atherosclerotic cardiovascular disease and increased Lp(a) levels [181]. The 2-phase trial (NCT05537571 sponsored by Silence Therapeutics (London, United Kingdom) for zerlasiran has already been completed in July 2024 [182], whereas the recruitment for the third phase of a clinical trial (NCT06292013, sponsored by Eli Lilly and Company (Indianapolis, IN, USA)) of lepodisiran (LY3819469) is in progress [183].

#### 3.3.1. Drug Manufacturing

**Figure 5:**
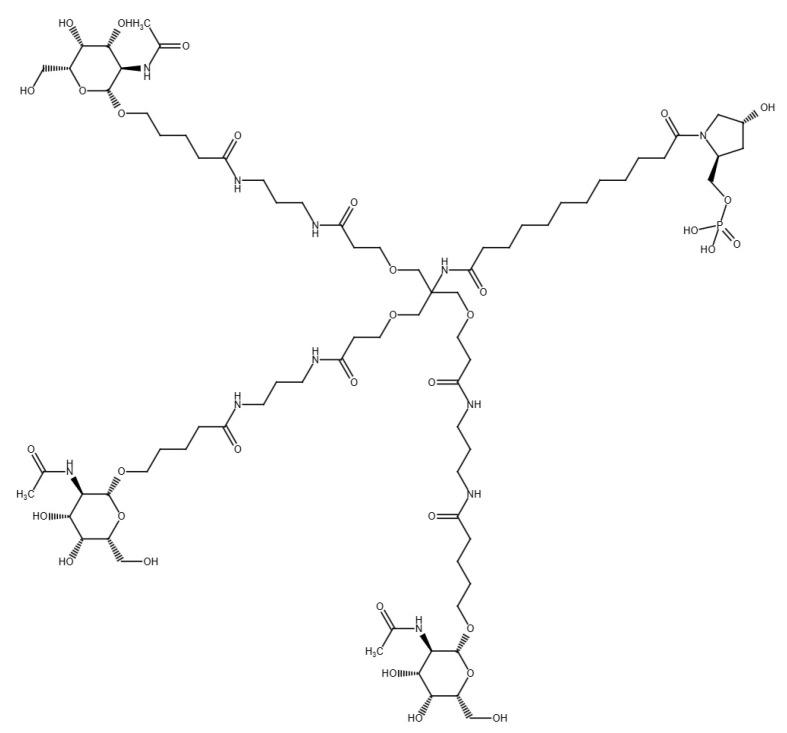
Structure of inclisiran based on Reaxys.

Inclisiran (Figure 5) is a structurally modified double-stranded siRNA. The sense strand of this drug is linked to a triantennary GalNAc ligand. The chemical modifications of inclisiran consist of modifications to the 2′-ribose, including 2′-*O*-methyl (32 nucleotides) and 2′-fluoro (11 nucleotides), and one ribose was replaced by a deoxyribose. In addition, a total of six phosphorothioate groups were added to the molecule, including two to the 5′ end of the sense strand and two groups each to the 5′ and 3′ ends of the antisense strand [184].

Olpasiran is a siRNA drug that has been covalently linked to triantennary GalNAc. Structural modifications to olpasiran involve the introduction of 2′-*O*-methylated and 2’-fluorinated nucleotides into the phosphorothioate molecule bonds. Thanks to these transformations, the molecule has a more stable chemical structure, which increases its resistance to degradation by nucleases [8].

Zerlasiran is a 19-nucleotide siRNA covalently coupled to GalNAc [185].

Lepodisiran is a 2-*O*-Me, 2′-F, and unmodified Dicer siRNA conjugated to three molecules of triantennary GalNAc, forming a tetraloop structure [186,187].

#### 3.3.2. Mechanism of Action and Pharmacological Effects

Small interfering RNA is a synthetic molecule consisting of two strands of RNA [173]. Once in the cytoplasm of the cell, the two strands are broken down into a sense strand and an antisense strand. In the next phase, the siRNA molecule is incorporated into the inducible RNA silencing complex (RISC), which is a multiprotein complex containing a nuclease. In its active form, RISC consists of a guide strand that binds precisely to the target mRNA sequence. As a result of the action of the argonaute-2 protein, the mRNA is cleaved, leading to degradation and consequent down-regulation of the protein [172,173].

The mechanism of action of inclisiran involves the degradation of PCSK9 mRNA. Conjugation with the GalNAc ligand enables liver cells to take up siRNA and successfully target PCSK9. As a result, PCSK9 is inactivated, leading to increased uptake of LDL-C and lower blood LDL-C levels [178].

#### 3.3.3. Pharmacokinetics of siRNA Drugs

##### Inclisiran

Inclisiran reaches a maximum concentration of 509 ng/mL within 4 h of subcutaneous administration. The medication has a large apparent volume of distribution of as much as 500 L. It is significantly (87%) bound to plasma proteins. The half-life of this siRNA-based drug is approximately 9 h, and its repeated use does not lead to accumulation in the body [179].

The ORION-1 randomized controlled trial involved 501 patients diagnosed with or at-risk of atherosclerotic cardiovascular disease and with high LDL-C levels. The use of inclisiran resulted in a significant decrease in LDL-C, on average 28–52% in patients without diabetes and 28–55% in patients with diabetes. It was also noted that patients treated with this drug had reduced levels of PCSK9, non-HDL cholesterol, Lp(a), and apoB, but increased concentration of HDL cholesterol [188]. The ORION-10 study included 1561 patients with atherosclerotic cardiovascular disease, while the ORION-11 study enrolled 1617 patients diagnosed with atherosclerotic cardiovascular disease or equivalent risk of such disease. All patients had elevated LDL cholesterol levels despite treatment with a statin at the maximum tolerated dose. After 510 days of inclisiran, LDL-C levels were measured in all participants. Patients in the ORION-10 study had an average 52.3% reduction in LDL-C concentration, while individuals in the ORION-11 trial exhibited an LDL-C reduction of 49.9% [189].

##### Olpasiran

Available data from a clinical trial (NCT04987320) [190] show that the maximum observed concentration of olpasiran after low-dose administration reached an average of 144 ng/mL, while after high-dose application it was as high as 549 ng/mL. The time required to achieve this concentration averaged 3 h. The apparent volume of distribution of olpasiran after low-dose administration was 238 L, while it was 176 L after high-dose administration [190].

The randomized double-blind phase 2 study involved 281 patients with atherosclerotic cardiovascular disease who had Lp(a) levels higher than 150 nmol/L. Study participants received various doses of olpasiran, including 10 mg, 75 mg, and 225 mg every three months, either 225 mg every six months, or a placebo. After 36 weeks, Lp(a) concentration was measured. The greatest decrease was achieved with the 225 mg dose administered for 12 weeks (−101.1%) and for 24 weeks (−100.5%). At the 10 mg dose, Lp(a) levels decreased by 70.5%, while a reduction of up to 97.4% was achieved with the 75 mg dose. In contrast, it was observed that patients who received a placebo had a 3.6% increase in Lp(a) levels [191].

##### Zerlasiran

The single ascending dose trial (phase 1) involved 32 participants with Lp(a) levels above 150 nmol/L and no cardiovascular disease. Patients were randomly divided into groups receiving either placebo or zerlasiran at doses of 30 mg, 100 mg, 300 mg, or 600 mg. The results of the study confirm that the reduction in Lp(a) concentration depends on the dose of zerlasiran used. The greatest reductions were observed at the 600 mg and 300 mg doses by 98% and 96%, respectively. The 100 mg dose showed a slightly lower reduction by 86%. Significantly weaker effect of −46% was achieved at the 30 mg dose. In patients taking a placebo, average Lp(a) levels decreased by only 10% [185].

##### Lepodisiran

The randomized dose-ascending clinical study (phase 1) included 48 participants with Lp(a) levels above 75 nmol/L and no cardiovascular disease. Patients were divided into groups receiving either placebo or lepodisiran at doses of 4 mg, 12 mg, 32 mg, 96 mg, 304 mg, or 608 mg. The results show that the extent of the reduction in blood Lp(a) levels depends on the dose of the drug used. The greatest decreases in concentration were observed at doses of 608 mg, 304 mg, and 96 m by 97%, 96%, and 90%, respectively. Significantly weaker results were achieved with doses of 4 mg (−41%), 12 mg (−59%), and 32 mg (−76%). In patients receiving placebo, mean Lp(a) levels were reduced by only 5% [186].

#### 3.3.4. Summary of Clinical Trials Comparing siRNA-Based Drugs with Other Classes of Lipid-Lowering Drugs

Inclisiran significantly reduced LDL-C compared to statins (mean −15.21, 95% CI [−25.19, −5.23]) but showed no significant difference from statin combined with ezetimibe. Inclisiran, compared to PCSK9 inhibitors, showed superiority, which may be due to its less frequent dosing regimen and longer time of observation in the studies [162]. Evolocumab reduced LDL-C by 61.09% (95% CI −64.81, −57.38), alirocumab reduced LDL-C by 46.35% (95% CI −51.75, −41.13), and inclisiran (284 mg) reduced LDL-C by 54.83% (95% CI −59.04, −50.62). Evolocumab and alirocumab demonstrated a significant reduction in the risk of major adverse cardiovascular events compared to inclisiran. Moreover, alirocumab reduced cardiovascular and all-cause mortality [192].

## 4. Safety of Antidiabetic and Antilipemic Drugs

Pharmacological treatment of diabetes and dyslipidemia significantly improves metabolic control and reduces the risk of cardiovascular disease. Unfortunately, it is also associated with potential side effects that may affect the patient’s quality of life and their decision to continue therapy.

It is imperative to be cognizant of the potential adverse effects associated with antidiabetic drugs, as these may have deleterious consequences for the patient’s health. The most common adverse effect of insulin and its analogues is hypoglycemia. Other adverse effects include weight gain, allergic reactions, lipoatrophy, and lipohypertrophy [193]. Due to their mechanism of action, incretin drugs (GLP-1 analogues, DPP-4 inhibitors) are characterized by side effects mainly related to the digestive system, such as nausea, vomiting, and loss of appetite [194,195]. Moreover, several serious adverse effects have been reported by patients following the administration of GLP-1 analogues. These include thyroid cysts, ketosis, and acute cholecystitis [194]. Furthermore, the most prevalent adverse effects associated with DPP-4 inhibitors encompass upper respiratory tract infections and nasopharyngitis [195]. It should be noted that DPP-4 inhibitors (lower dose) can be used to treat patients with moderate to severe renal impairment. In contrast, the use of GLP-1 analogues is contraindicated in these cases [196]. SGLT-2 inhibitors increase urinary glucose excretion, which can lead to infections of the urinary and reproductive tracts. Furthermore, orthostatic hypotension can be a side effect due to increased diuresis. Another reported complication is ketoacidosis. The main contraindication to using SGLT-2 inhibitors is severe renal failure [97,99,100].

The use of hypolipemic drugs generally results in mild adverse effects. However, in some cases, these medications can lead to serious complications. It is imperative to know the safety profile of a drug before the initiation of therapeutic intervention. Inhibitors of PCSK9 most commonly cause reactions at the injection site, such as pain, redness, or swelling. These medications can also lead to flu-like symptoms, eczema, and upper respiratory tract infections [153,156]. Among ASOs, volanesorsen is an approved therapeutic agent with a well-characterized safety profile. The most prevalent adverse effects of this drug are injection site reactions and thrombocytopenia, which is also a contraindication for the use of this drug. Serious adverse effects reported by patients include liver and kidney dysfunction [168]. Currently, pelacarsen is undergoing clinical trials; consequently, data concerning the safety profile of this drug are limited. Data from the clinical trials indicate that the most common adverse effect of pelacarsen is injection site reactions [169,170]. As with antisense nucleotides, there is limited safety data on siRNA-based drugs. Of this group of drugs, inclisiran is the only one authorized, and its safety profile is documented. The most common side effects of inclisiran are injection site reactions [179]. Olpasiran, zerlasiran, and lepodisiran are currently in clinical trials, and available data suggest that the most common side effects of these drugs are also injection site reactions [186,191,197].

The above data are summarized in Table 5.

## 5. Discussion

Emerging biopharmaceuticals are engineered to target specific molecular pathways, providing innovative therapeutic strategies that overcome the limitations of conventional treatments [198]. The strategy of drug development presents significant potential; however, its limited success can be attributed, in part, to insufficient therapeutic efficacy. Effectiveness in basic research and early phases of clinical trials determines whether findings from the efficacy studies are applicable in a typical community. In the case of metabolic syndrome, the primary challenges are achieving good glucose control, maintaining a normal body weight, and preventing diabetes complications.

The process of translation from scientific discoveries into practical applications is known as translational research or commonly named “bench to bedside”. It comprises two phases. In the first phase, basic research begins translation discoveries at “the bench” by studying disease at a molecular or cellular level. Progress to the clinical level means moving to the patient’s bedside. There is a growing recognition within the scientific community that translational research follows a bidirectional pathway, whereby insights from clinical practice inform basic science, just as discoveries at the bench guide clinical applications. In the second phase, the adoption of promising clinical research by community-based healthcare systems under uncontrolled and often uncontrollable conditions is promoted. The effectiveness, dissemination, and implementation research, as well as policy research, are particularly considered [199].

Overcoming the barriers to translating scientific advances into clinical practice is a challenge. The integration of advances in delivery technologies, biomarker-driven patient stratification, and longitudinal clinical studies is required to establish efficacy and safety in diverse populations. An accelerated pathway to clinical trials should be grounded in a collaborative framework between academic researchers and manufacturers operating in compliance with Good Manufacturing Practice (GMP) standards. The production of biological drugs frequently utilizes cutting-edge biotechnological methods and novel types of biological therapeutics such as cellular or genetic biologics, which constitute the limitations of biomedical research. Thus, the manufacturing process of most biological drugs is extremely complex. Regulatory and manufacturing challenges persist, particularly regarding batch consistency, scalability, and cost effectiveness [200].

Biopharmaceuticals are highly specific and characterized by very low toxicity. However, they may exhibit immunogenicity in some patients, unlike most small-molecule drugs. In certain patients, biopharmaceuticals may elicit immune responses that contribute to a gradual and unpredictable reduction in therapeutic efficacy, accompanied by an increase in immunogenicity [200].

Despite the therapeutic promise of nucleotide-based modalities such as siRNAs and ASOs, their clinical translation in the context of metabolic disorders remains constrained by several critical challenges. To begin with, efficient and tissue-specific delivery systems are still under active development. The primary route of administration is still intravenous application. The physiological complexity and systemic nature of metabolic disorders require targeting beyond hepatocytes, which currently represent the primary locus for successful oligonucleotide delivery.

Second, the biopharmaceuticals are usually unstable, and a wide range of specific enzymes, such as nucleases, peptidases, proteinases, and hydrolases, are engaged in their metabolism. Moreover, the pharmacokinetic and pharmacodynamic profiles of agents like ASO often necessitate chemical modifications such as 2′-methoxyethyl or 2′-*O*, 4′-*C*-methylene-bridged nucleic acid, which enhance resistance to nuclease degradation and bioavailability while minimizing off-target effects and immunogenicity. Moreover, a greater structural similarity between therapeutic nucleic acids and other RNA-targeting molecules may increase the risk of off-target interactions and unintended adverse effects [201]. A key advantage of oligonucleotide-based therapeutics is that their delivery efficiency and pharmacodynamic activity are primarily dictated by chemical modifications. On the other hand, their target specificity is conferred by the complementary nucleotide sequence. Chemical design of oligonucleotides enables appropriate distribution and safety profiles for clinical gene silencing in a particular tissue. The modifications can inadvertently alter the therapeutic index. To achieve clinical productivity, the chemical structure of oligonucleotides is optimized with a combination of sugar, backbone, nucleobase, and 3′- and 5′-terminal modifications [202]. For this reason, we were mostly focused on the chemical modifications of biological drugs, which are not so widely described in the literature as their clinical efficacy.

Last but not least, the heterogeneity of metabolic disorders and the influence of lifestyle and genetic background complicate the design of universally effective oligonucleotide therapies. Long-term safety data remain limited regarding chronic administration, which is likely to be essential to the sustained management of metabolic disorders.

## 6. Future Perspective

The development of biopharmaceuticals for metabolic syndrome and its comorbidities is advancing rapidly, driven by innovations in molecular targeting, drug delivery systems, and personalized medicine. The emerging therapies, their mechanisms, clinical potential, and integration are still a challenge. The market for therapeutics for metabolic disorders is expected to grow at a Compound Annual Growth Rate (CAGR) of 7.8%, to reach USD 213 billion by 2037 [203,204]. Some drugs, such as tirzepatide, a dual agonist of GIP and GLP-1 receptors, have been approved by the FDA for the treatment of T2D. Moreover, they have demonstrated unprecedented efficacy in clinical trials targeting obesity. Preventive care and personalized strategies should therefore be introduced. Unfortunately, high prices of biologics limit accessibility in low-resource settings. Scaling up global access to advanced therapies while addressing cost barriers remains critical. In addition, biomarker-driven approaches (e.g., genetic profiling for gene therapy) require infrastructure investment. There is no doubt that precision and personalized medicine should be based on genetic therapies and receptor-specific agents, which could enable curative approaches.

More efforts should be made to develop new ways of delivering biological drugs, such as hydrogels, which will facilitate their use. Given the multifactorial and complex nature of the metabolic syndrome, the use of combination therapies warrants careful consideration to achieve comprehensive and effective treatment. Dual-target agents (e.g., GLP-1/GIP agonists) and hydrogel-based multidrug delivery are likely to dominate next-generation pipelines. The major direction for the further development of biological drugs is research into alternative, less invasive routes of administration, particularly oral. Subcutaneous administration of drugs, although widely used, is often resisted and feared by patients, both due to the discomfort associated with injection of the drug and possible side effects. The injectable form of the drug can be a barrier to regular use and thus lead to ineffective therapy. For this reason, the development of oral forms of biological drugs is crucial to improve patient comfort. This evolving landscape positions biopharmaceuticals as central to redefining the management of metabolic syndrome, with a shift from symptomatic treatment to disease modification and prevention.

## 7. Conclusions

Biological therapies are driving a significant shift in modern medicine. Their contribution to the treatment of metabolic syndrome has been outstanding despite a relatively short time of clinical usage. Continued effort is being made in the development and refinement of biological drug modifications. Incorporation of fatty acid moieties into polypeptide-like drugs, methyl, methoxyethyl, or fluor substitution in nucleic acids enables fine-tuning the properties of molecules to specific targets, as well as improving the pharmacokinetic properties. The primary challenge appears to be the early identification of potential adverse side effects in the early stages of clinical trials. A secondary goal is to improve the routes of administration, since subcutaneous injections, although widely used, may be poorly tolerated or unacceptable to certain patients. The need for frequent injections limits patient compliance. New peptide- or nucleotide-based drugs in new formulations are therefore highly anticipated.

## Figures and Tables

**Table 1:** Types of insulin and their action time [23,24,25,26,27].

Insulin Type *	Preparation	Action Onset	T_max_	Time Range
rapid-acting insulin	aspart (NovoLog)	15 min	1–3 h	3–5 h
	glulisine (Apidra)	12–30 min	1.5 h	5–6 h
	lispro (Admelog, Humalog, Lyumjev)	15–30 min	0.5–2 h	2–5 h
ultra-rapid acting insulin	aspart (Fiasp)	5 min	0.5 h	3–5 h
regular or short-acting insulin	human regular (Humulin R, Novolin R, Velosulin R)	0.5–1 h	2–4 h	5–8 h
intermediate-acting insulin	isophane/NPH (Humulin N, Novolin N, ReliOn)	2–4 h	4–10 h	8–16 h
long-acting insulin analogue	detemir (Levemir)	1–2 h	6–12 h	20–24 h
	glargine (Basaglar, Lantus)	2–4 h	peakless	24 h
	degludec (Tresiba)	0.5–1.5 h	peakless	42 h

* Units: 100 U/mL.

**Table 2:** Complex, biphasic insulins (insulin mixtures).

Insulin Type in the Mixture	The Content of Insulin, Long- or Short-Acting
insulin aspart with protamine suspension of insulin aspart (analogue mixture)	30% or 50%
insulin aspart with insulin degludec (dual analogue mixture)	30%
insulin lispro with protamine insulin lispro (analogue mixture)	25% or 50%
biphasic insulin, human	20%, 25%, 30%, 40%, 50%

**Table 3:** Anti-diabetic drugs: summary of clinical studies and pharmacological effects.

Drug	Study Acronym	Pharmacological Effects	References
GLP-1 analogues			
Dulaglutide	REWIND	lowering systolic BP ^1^ (−1.7 mmHg) compared to placebo; mean reduction in HbA_1c_ ^2^ −0.61% vs. placebo	[70]
Exenatide	-	lowering systolic BP (−1.57 mmHg) compared to placebo; increased diastolic BP (+0.25 mmHg)	[73]
Lixisenatide	ELIXA	moderate and persistent difference in systolic BP between the lixisenatide-treated and the placebo groups (−0.8 mmHg in favor of lixisenatide); HbA_1c_ ^2^ −0.6% vs. placebo −0.2%	[76]
Liraglutide	SCALE Obesity and Prediabetes	weight loss (−8.4 ± 7.3 kg); weight loss of at least 5% (63.2% vs. 27.1%), more than 10% (33.1% vs. 10.6%), and more than 15% (14.4% vs. 3.5%) compared to the placebo; lowering systolic BP (−4.2 ± 12.2 mm Hg study group vs. −1.5 ± 12.4 placebo group) and diastolic BP (−2.6 ± 8.7 mm Hg study group vs. −1.9 ± 8.7 placebo group); lowering HbA_1c_ (−0.30 ± 0.28% vs. placebo −0.06 ± 0.30%), glucose (−7.1 ± 10.8 mg/dL vs. placebo + 0.1 ± 10.4 mg/dL), and fasting insulin (−12.6% vs. placebo −4.4%) levels	[72]
	LEADER	lowering systolic BP (−1.2 mmHg) after 36 months compared to placebo; increased diastolic BP (+0.6 mmHg) compared to placebo	[74]
	LEAN	improved liver and adipose insulin sensitivity by augmenting insulin-mediated inhibition of lipolysis; reduction in lipotoxic metabolites and pro-inflammatory mediators; reduced lipogenesis in the liver (−1.26% vs. placebo + 1.30%)	[71]
Semaglutide		reduction in caloric intake (24%, −3036 kJ) and better control of eating weight loss (12-week semaglutide therapy: −5.0 kg vs. placebo: +1.0 kg) appetite and fat reduction	[54]
	SUSTAIN-6	0.5 mg dose: decreased systolic BP (−3.4 mmHg vs. −2.2 mmHg placebo); HbA_1c_ −1.1% vs. placebo −0.4%; 1.0 mg dose: decreased systolic BP (−5.4 mmHg vs. −2.8 mmHg placebo), HbA_1c_ −1.4% vs. placebo −0.4%; comparable diastolic BP	[75]
		enhanced glucose-dependent insulin secretion; increased the sensitivity of pancreatic β-cells to glucose; more efficient insulin secretion in response to elevated blood glucose levels; reduction in glucagon levels; reduced fasting and postprandial blood sugar levels	[77]
DPP-4 inhibitors			
Alogliptin	EXAMINE	lowered HbA_1c_ vs. placebo; no increased risk of cardiovascular incidents	[78]
Linagliptin	CAROLINA	increased risk of cardiovascular incidents	[79]
Saxagliptin	SAVOR-TIMI 53	lowered fasting glucose levels vs. placebo after 2 years and at the end of therapy (*p* < 0.001); lowered HbA_1c_ vs. placebo; a higher proportion of patients in the saxagliptin group achieved HbA_1c_ < 7% at the end of the treatment period (36.2% vs. placebo 27.9%); no effect on ischemic events	[80]
Sitagliptin	TECOS	after 4 months of treatment, HbA_1c_ −0.4% vs. placebo; no effect on cardiovascular risk	[81]
Vildagliptin	-	no increased risks of hospitalization for heart failure, non-fatal stroke, non-fatal myocardial infarction, and cardiovascular death	[82]
SGLT-2 inhibitors			
Canagliflozin	CANVAS	slowed eGFR ^3^ decline in patients with various causes of CKD, including those without diabetes	[83]
	CREDENCE	34% reduction in the risk of death from renal causes and a 32% decrease in the risk of ESRD ^4^	[84]
Dapagliflozin	-	19% reduction in EAT ^5^ thickness; inhibition of glucose uptake (21.6% inhibition of 2-deoxy-2-[18F]fluoro-D-glucose (FDG))	[85]
	DAPA-CKD	slowed eGFR decline in patients with various causes of CKD, including those without diabetes	[86]
Empagliflozin	EMPA-REG OUTCOME	reduction in the primary composite cardiovascular outcome and all-cause mortality	[87]
	EMPA-REG OUTCOME	an initial transient decrease in eGFR is commonly observed; with long-term therapy eGFR values tend to increase after treatment discontinuation	[88]

^1^ BP, blood pressure; ^2^ HbA_1c_, glycated hemoglobin; ^3^ eGFR, estimated glomerular filtration rate; ^4^ ESRD, end-stage renal disease; ^5^ EAT, epicardial adipose tissue.

**Table 4:** Pharmacokinetic characteristics of drugs used in T2D.

Drug	t_1/2_	T_max_	C_max_	V_d_	References
GLP-1 analogues					
Dulaglutide	5 days	48 h	114 ng/mL	3.09 ^a^, 5.98 L ^b^	[61]
Exenatide	2.4 h	2 h	211 pg/mL	28 L	[59,89]
Lixisenatide	3 h	1- 3.5 h	N/A ^c^	100 L	[58]
Liraglutide	13 h	8–12 h	9.4 nmol/L (0.6 mg)	11–17 ^d^ or 20–25 ^e^ L	[57,90]
Semaglutide	7 days	1–3 days	N/A	12.4 L	[62,91]
DPP-4 inhibitors					
Alogliptin	21 h	1–2 h	N/A	417 L	[92]
Linagliptin	12 h	1.5 h	N/A	1110 L	[93]
Saxagliptin	2.5 h	2 h	24 ng/mL	N/A	[94]
Sitagliptin	12.4 h	1–4 h	950 nM	198 L	[95]
Vildagliptin	3 h (p.o.); 2 h (i.v.)	1.7 h	N/A	71 L	[96]
SGLT-2 inhibitors					
Canagliflozin	10.6 ± 2.13 h (100 mg)		1.059 ng/mL (100 mg)		[97,98]
	13.1 ± 3.28 h (300 mg)	1–2 h	2.792 ng/mL (300 mg)	83.5 L	
Dapagliflozin	12.9 h	2 h	158 ng/ml	118 L	[99]
Empagliflozin	12.4 h	1.5 h	259 nmol/L (10 mg) 687 nmol/L (25 mg)	73.8 L	[100]

^a^ The apparent population mean central volume of distribution. ^b^ The apparent population mean peripheral volume of distribution. ^c^ Not available; ^d^ Victoza^®^; ^e^ Saxenda^®^.

**Table 5:** Side effects of antidiabetic and antilipemic drugs.

Group of Drugs	Adverse Drug Reactions	References
Antidiabetic drugs		
Insulin and insulin analogues	hypoglycemia, weight gain, lipodystrophy	[193]
GLP-1 analogues	nausea; vomiting; loss of appetite; thyroid cysts ^a^; ketosis ^a^; acute cholecystisis ^a^	[194]
DPP-4 inhibitors	nausea, vomiting; loss of appetite, upper respiratory tract infections; nasopharyngitis	[195]
SGLT-2 inhibitors	infections of the urinary and reproductive tracts, dehydration, orthostatic hypotension; ketoacidosis	[97,99,100]
Antilipemic drugs		
PCSK-9 inhibitors	injection site reactions; flu-like symptoms; eczema; upper respiratory tract infections	[153,156]
Antisense oligonucleotides	injection site reaction; trombocytopenia ^b^; liver dysfunction ^a,b^; kidney dysfunction ^a,b^	[168,169,170]
siRNA-based drugs	injection site reactions	[179,186,191,197]

^a^ rarely, ^b^ adverse effects of volanesorsen.

## Data Availability

No new data were created or analyzed in this study.
